# CB2 and TRPV1 receptors in inflammatory state of macrophages from sickle cell anemia pediatric/young adults

**DOI:** 10.1038/s41598-025-15028-2

**Published:** 2025-08-08

**Authors:** Giuseppe Di Feo, Giulia Giliberti, Deeksha Rana-Seyfert, Maria Maddalena Marrapodi, Maddalena Casale, Shakeel Ahmed, Silverio Perrotta, Francesca Rossi, Domenico Roberti, Alessandra Di Paola

**Affiliations:** 1 Department of Woman, Child and General and Specialist Surgery, University of Campania “Luigi Vanvitelli”, Napoli, 80138 Italy; 2 Department of Advanced Medical and Surgical Sciences, University of Campania “Luigi Vanvitelli”, Napoli, 80138 Italy; 3 Department of Life Science, Health and Health Professions, Link Campus University, Rome, 00165 Italy

**Keywords:** Sickle cell anemia, Inflammation, Macrophage polarization, Iron metabolism, Endocannabinoid/Endovanilloid system, Inflammation, Cell biology, Cell polarity, Haematological diseases, Sickle cell disease

## Abstract

Sickle Cell Disease (SCD) is a monogenic disorder characterized by the production of abnormal hemoglobin. Polymerization of HbS causes sickling of red blood cells (RBCs) evidenced by acute adverse events and persistent inflammatory state, vasculopathy and organ damage. Sickled RBCs cause an anemic condition and vaso-occlusive crisis which trigger leukocytes, endothelial cells, and platelets. Due to these events, SCD patients unveiled an elevated level of pro-inflammatory cytokines, which contribute to the ongoing inflammatory state, oxidative stress, and other severe complications. SCD patients also experience neuropathic, inflammatory, and nociceptive pain. The discovery of novel therapeutic approaches and targets to counteract and manage inflammation in SCD are needed. Our study aimed to better understand the role of macrophages in SCD inflammation by first investigating their phenotype and then studying the iron metabolism involvement in the inflammatory processes. Therefore, given the importance to find novel therapeutic approach to contain and manage inflammation in these patients, and considering the role of CB2 and TRPV1 in this process, we decided to investigate the expression of these receptors and the effects of their stimulation on inflammatory state in SCD macrophages.

**Supplementary Information:**

The online version contains supplementary material available at 10.1038/s41598-025-15028-2.

## Introduction

Sickle cell disease (SCD) is a monogenic hemoglobinopathy with approximately 515,000 affected children born every year^1^.

SCD is caused by a missense mutation in β-globin gene responsible for the production of a hemoglobin variant called hemoglobin S (HbS), prone to polymerization to form fibers that alter red blood cells (RBCs) rheology and causing their sickling and, consequently, RBCs impaired function. Homozygosity for the HbS allele is clinically referred as sickle cell anemia^2^. The main clinical manifestations of SCD are represented by anemia, vaso-occlusive crisis (VOCs), oxidative stress, organ damage, inflammation and pain development^3,4^, leading to multiorgan damage^5^. Management of SCD patients requires a multi-disciplinary approach. Vaccines and antibiotics are used to prevent and treat infections; other drugs, such as hydroxyurea, L-glutamine (ENDARI) and crizalizumab (Adakveo^®^) (both only in the US) are used to increase hemoglobin (Hb) levels, prevent VOCs and strokes as well as manage acute events. Specifically, hydroxyurea acts on many disease-related features SCD: it increases HbF counteracting HbS polymerization, with mechanisms of action still incompletely understood, carries cytotoxic effects reducing marrow production of neutrophils, reticulocytes and platelets reducing the inflammation-derived risk of VOCs, and induces, as said, beneficial effect on circulating erythrocytes, increasing MCV and Hb levels^6^. L-Glutamine, instead, is one of the most abundant amino acids and it is required to synthesize NAD. Treatment with L-glutamine increases the NAD redox ratio and reduces adhesion of sickle RBC to endothelial cells, a hallmark of vaso-occlusive painful crises^7^. ENDARI was approved by FDA since 2017, while the Sponsor withdrew their EMA marketing authorization request because of change in the company’s marketing strategy but, at the time of the withdrawal, the Agency was of the opinion that the marketing authorization for the treatment sickle cell disease should be refused because the main and the supportive studies did not show the compound to be effective at reducing the number of sickle cell crises or hospital visits mostly because a large number of patients dropped out of the study before it was finished, and information on how the medicine worked for those patients was not available as well as concomitant treatments with Hydroxyurea was not well balanced among groups in the supportive study.

^8^. Crizanlizumab, instead, is a monoclonal antibody that binds to P-selectin on endothelial cells and platelets, preventing their interaction with P-selectin glycoprotein ligand 1 on endothelial cells, platelets, red blood cells, and leukocytes^9^ and was firstly approved by FDA in November 2019 and remains on the market in the U.S. The EMA and MHRA marketing authorizations for Adakveo^®^, initially released in 2020, were revoked by the agencies as the confirmatory STAND study (NCT03814746)^10^ did not demonstrate a significant difference between crizanlizumab and placebo in annualized rates of VOCs.

Additionally, many SCD patients undergo RBC transfusion and/or red cell exchange, which may lead to iron overload and elevated hepcidin levels^1,11^. By adulthood, nearly 90% of SCD patients often show impaired iron homeostasis^12,13^. Iron overload represent the one of the side effects of blood transfusion requiring targeting treatment. Several researches indicate that SCD patients often show impaired iron homeostasis driven by multifactorial mechanisms regulating hepcidin levels^11^.

It is known that iron has a key role in different metabolic processes and that the dysregulation of its metabolism can be cause inflammation and consequently cell damage^14^. Interestingly, high levels of pro-inflammatory cytokines, particularly IL-6, stimulate hepcidin production and release, which binds to ferroportin 1 (FPN-1) - the only known cell iron exporter - leading to iron sequestration within macrophages^15,16^. Inflammation represent another crucial factor in SCD pathogenesis^4^, indeed in SCD patients are reported high levels of pro-inflammatory cytokines which contribute to a persistent inflammatory state^17^. Macrophages represent the main cell type involved in inflammation. Particularly, they show two different main functional and structural phenotypes: classically activated M1 macrophages or alternatively activated M2 ones^15,18–20^. M1 macrophages show pro-inflammatory, anti-cancer and anti-microbial properties. They can be activated by Interferon-gamma (IFN-γ), Tumor Necrosis Factor-alpha (TNF-α) and Bacterial Lipopolysaccharide (LPS) and are characterized by the release of pro-inflammatory cytokines, such as Interleukin (IL)−6, Interleukin (IL)−1β, Tumor Necrosis Factor (TNF-α) and inducible Nitric Oxide Synthase (iNOS). Conversely, M2 macrophages exert anti-inflammatory and immuno-suppressive effects. They release anti-inflammatory cytokines, like IL-10 and Transforming Growth Factor (TGF)-β and are activated by IL-4, IL-10, IL-13, and the mTOR-PI3K-Akt signaling pathway activation^15,18–20^.

Macrophages play an important role in modulating iron homeostasis^21^. In particular, M1 macrophages are involved in iron internalization, which is also correlated to a marked increase of protein expression levels of divalent metal transporter-1 (DMT1); conversely, M2 phenotype is responsible for iron release. The increase of intracellular iron concentration causes an impairment of their activities and functions, thus contributing to inflammatory processes^15,18–20^.

As previously mentioned, a severe complication in SCD patients is represented by acute and chronic daily pain, linked to chronic ischemia-reperfusion injury, repeated vascular occlusion, and inflammation^22–24^. SCD pain is identified as nociceptive, neuropathic, or inflammatory pain^23^. Several anti-inflammatory drugs or opioids are used for pain treatment in SCD^23^. Therefore, there is great interest in finding novel therapy to counteract SCD pain, targeting other pathways.

The endocannabinoid system (ECS) is an endogenous system involved in the regulation of homeostasis, inflammatory and immune responses, internal environment balance, and pain^25^. It is constituted by the Cannabinoid Receptor Type 1 (CB1) and Type 2 (CB2), their endogenous ligands and all the enzymes involved in their biosynthesis and degradation^25,26^. CB1 is localized in central nervous system, while CB2 is mainly expressed by peripheral cells, in particular by immune cells (macrophages and lymphocytes). CB2 is a mediator of anti-inflammatory and immune-modulating properties of several cell types^20,27^, and it is involved in the pathogenesis of several autoimmune and inflammatory diseases, by exhibiting a reduction of its expression^18,20,26–29^.

Cannabinoids receptors are involved in inflammation and in neuropathic pain in mice with SCD and their stimulation with agonists modulates mast cell activity and neuroinflammation also reducing pain^30–32^. Interestingly, the use of cannabinoids in SCD patients to counteract pain has been tested^33–35^; nevertheless, the mechanisms at the basis of their analgesic function are not still clear^33^. Particularly, CB2 exerts neuroprotective properties in microglia^36^; indeed, in physiological conditions these cells show very low levels of CB2 receptor, while – under pro-inflammatory stimuli or during inflammatory and neuropathic pain – CB2 levels increase^36–38^. It seems that this variation of CB2 levels is a protective reaction of microglia to counteract the inflammatory responses and to ameliorate pain^39^. The stimulation of CB2 with selective agonists inhibits both pro-inflammatory processes and neuropathic pain development^39^. Interestingly, it is already reported that cannabinoid receptors are involved in inflammation and in neuropathic pain in mice models of SCD and their stimulation is able to contain both aspects.

Another receptor with an important role both in inflammation and in neuropathic pain is the transient receptor potential vanilloid 1 (TRPV1), a non-specific cation channel which can be activated by several stimuli (chemical, mechanical and thermal)^40^. TRPV1 is involved in pain development in several diseases, highlighting its capability to reduce or inhibit pain^41–44^. Particularly, it has been demonstrated that TRPV1 is upregulated and involved in peripheral sensitization in mice with SCD^45,46^. However, these data about TRPV1 in SCD have not yet been reported in humans^45^. Interestingly, considering the role of TRPV1 in SCD pain development, in a pilot safety and feasibility trial (SPICE) it has been tested high-dose (8%) topical capsaicin in pediatric patients with SCD and its-associated neuropathic pain to better clarify the role of capsaicin in pain modulation^45^. TRPV1, like CB2, is also involved in containing inflammatory processes. Several studies conducted in animal models revealed the potential of its stimulation to reduce inflammatory state^45,46^. Indeed, the stimulation of TRPV1 is responsible for the modulation of immune cells functions, in particular lymphocytes and macrophages^47^, indeed it has been demonstrated that this receptor is also involved in the polarization of macrophages, promoting the phenotype switch towards M2 anti-inflammatory^48^.

Therefore, CB2 and TRPV1 could be considered an attractive therapeutic target to counteract several inflammatory conditions, neurodegenerative disorders, alteration of bone metabolism, obesity, and pain^26^.

Since macrophages express both CB2 and TRPV1 receptors^18,20,49^, a better understanding of their involvement in inflammatory responses and in pain development could be interesting to manage the altered mechanisms existing also in SCD patients. Therefore, considering the strong correlation between iron and inflammation, and macrophages role in inflammatory responses, we firstly characterized phenotypes of macrophages isolated from peripheral blood of SCD patients and iron metabolism in these cells. We investigated the expression of CB2 and TRPV1 in SCD macrophages to highlight their possible involvement in the pathobiology of SCD-associated inflammation, and we evaluated the effects of these receptors’ stimulation on macrophage polarization and iron metabolism modulation to regulate and contain the inflammatory state in SCD patients.

## Results

### Characterization of macrophages isolated from SCD patients

Protein expression of M1 and M2 macrophages markers was assessed by Western Blots to characterize the macrophage phenotype in SCD patients.

**Fig. 1:**
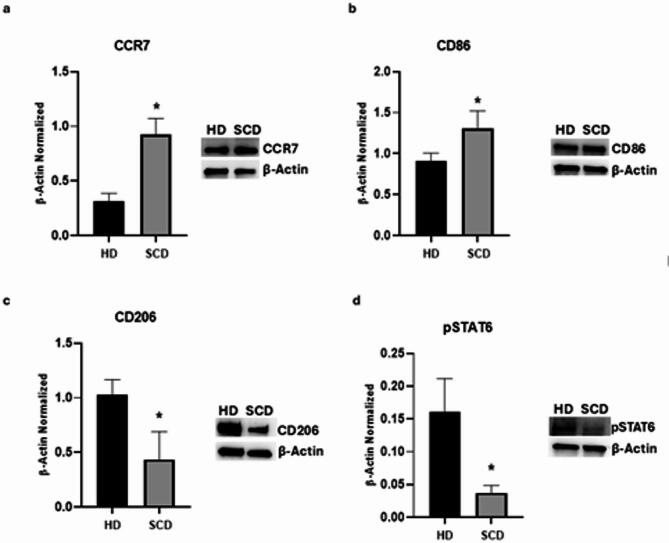
Characterization of macrophages derived from SCD pediatric/young patients. CCR7 (**a**), CD86 (**b**), CD206 (**c**), and pSTAT6 (**d**) protein expression levels in sickle cell disease (SCD) macrophages compared to those of healthy donors (HD), evaluated by Western blotting starting from 15 µg of total lysates. The most representative images are displayed. The protein bands were detected through Image Lab Ink 6.1 software “BIORAD”, and the intensity ratios of immunoblots compared to HD, taken as 1, were quantified after normalizing with the respective controls. The relative quantification for these proteins, normalized for the housekeeping protein β-Actin, is represented in the histograms as the mean ± SD. An unpaired t-test was performed for statistical analysis. *, *p* ≤ 0.05 compared to HD. Original blots/gels are presented in Supplementary Uncropped WB Figures.

Biochemical analysis revealed that SCD patients showed higher levels of M1 phenotype markers, CCR7 and CD86, compared to healthy donors (HD) (*p* = 0,0029 and *p* = 0,045, respectively), with a large effect size for CCR7 (η^2^ = 0,9135) and a moderate but relevant effect size for CD86 (η^2^ = 0,6742). The 95% of confidence interval (CI) of the mean difference ranged from 0,3506 to 0,8738 for CCR7 and from 0,01385 to 0,7783 for CD86 (Fig. 1A, B). Moreover, we also observed a concomitant reduction of the M2 phenotype marker CD206 and of pSTAT6, a protein involved in macrophages switching process from pro-inflammatory M1 phenotype to anti-inflammatory M2 one, in SCD macrophages compared to HD ones (*p* = 0,0231 and *p* = 0,0154, respectively), with a large effect size (η^2^ = 0,7626 and η^2^ = 0,8042, respectively; 95% CI [−1,056; −0,1342] and [−0,2072; −0,03875], respectively) (Fig. 1C, D).

These results indicate that in pediatric/young adults SCD patients there is a prevalence of M1 macrophage polarity which might contribute to the impairment of the inflammatory state and pain in SCD patients.

### Inflammatory profile of SCD macrophages

We performed several ELISA assays to investigate inflammatory cytokines released by SCD and HD macrophages. We observed significant elevated levels of the pro-inflammatory cytokines IL-6 and TNF-α in SCD macrophages compared to HD macrophages (*p* = 0,0067 and *p* = 0,0058, respectively), with a very large effect size (η^2^ = 0,87 and η^2^ = 0,88, respectively; 95% CI [498,2; 1662] and [2,33; 7,31], respectively). (Supplementary Figure S1 A, B). While, evaluating the anti-inflammatory cytokine IL-10 levels, we did not observe any significant variation in SCD samples compared to HD ones (Supplementary Figure S1 C).

These results support an impairment of the inflammatory state of SCD patients with a prevalence of pro-inflammatory state.

### Iron metabolism of SCD macrophages

Iron metabolism was evaluated assessing the hepcidin levels and intracellular iron concentrations, and the protein expression levels of the iron transporters transferrin receptor (TfR1), DMT1, and ferroportin (FPN-1).

**Fig. 2:**
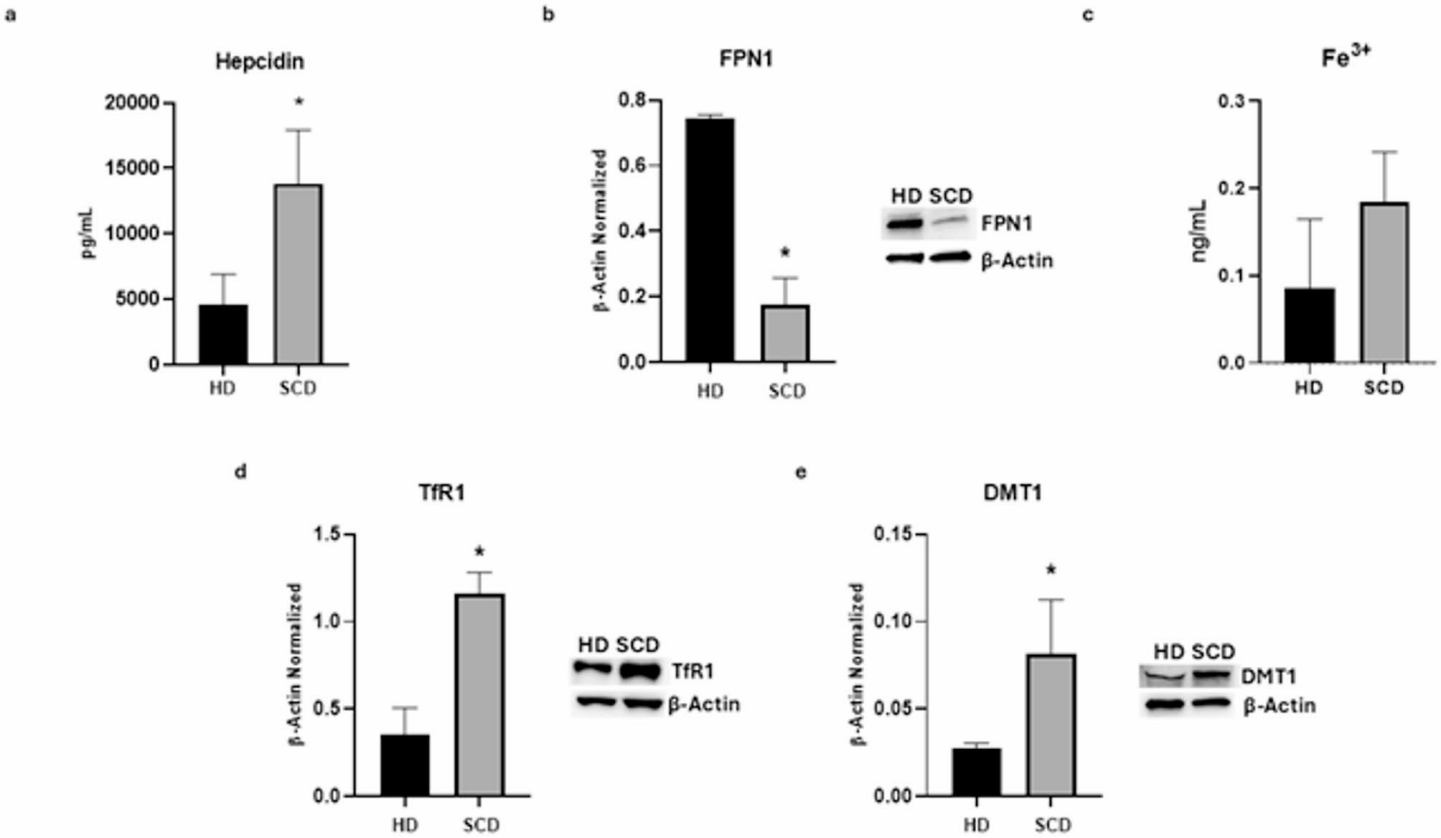
Iron metabolism in macrophages obtained from SCD pediatric/young patients. Evaluation of hepcidin (**a**) release from sickle cell disease (SCD) macrophages compared to healthy donors’ (HD) macrophages, revealed through an enzyme-linked immunosorbent assay (ELISA). The graphs show hepcidin levels (pg/mL) as the mean ± standard deviation (SD). An unpaired t-test was performed for statistical analysis. FPN1 (**b**), TfR1 (**d**), and DMT1 (**e**) protein expression levels in SCD macrophages of pediatric/young patients’ compared to HD, evaluated by Western blotting, starting from 15 µg of total lysates. The most representative images are displayed. The protein bands were detected through Image Lab Ink 6.1 software “BIORAD”, the intensity ratios of immunoblots compared to CTR, taken as 1, were quantified after normalizing with the respective controls. The relative quantification for these proteins, normalized for the housekeeping protein β-Actin, is represented in the histograms as the mean ± SD. An unpaired t-test was performed for statistical analysis. Original blots/gels are presented in Supplementary Uncropped WB Figures. Fe3+ (**c**) intracellular concentrations (nmol/µL) in HD and SCD macrophages of pediatric/young patients’, determined by iron assay. Histogram shows Fe3 + concentration as the mean ± SD. An unpaired t-test was performed for statistical analysis. * *p* ≤ 0.05 compared to HD.

As expected, we observed a statistically significant increase of hepcidin levels in SCD patients compared to HD (*p* = 0,027), with a large effect size (η^2^ = 0,74; 95% CI [1703; 16683]) (Fig. 2A). This result matches the strong increase of IL-6 levels in SCD macrophages (Supplementary Figure S1A). In line with the elevated hepcidin levels, we noticed a reduction of FPN-1 protein expression levels (*p* = 0,0003), with a large effect (η^2^ = 0,9724; 95% CI [−0,4350; 0,7002] (Fig. 2B). Moreover, we observed an increased expression of both iron importer, TfR1 and DMT1 (*p* = 0,0020 and *p* = 0,037, respectively), with a large effect size (η^2^ = 0,9283 and η^2^ = 0,7007, respectively: 95% CI [0,4927; 1,111] and [0,005054; 0,1041], respectively) (Fig. 2D, E). We also highlighted a trend to increase of intracellular iron concentration, even though not in statistically significant manner in SCD patients compared to HD (*p* = 0,1546), with a large effect size (η^2^ = 0,4343; 95% CI [−0,05744 to 0,2540]), indicating uncertainly due to the small sample size (Fig. 2C).

### CB2 and TRPV1 expression in SCD macrophages

**Fig. 3:**
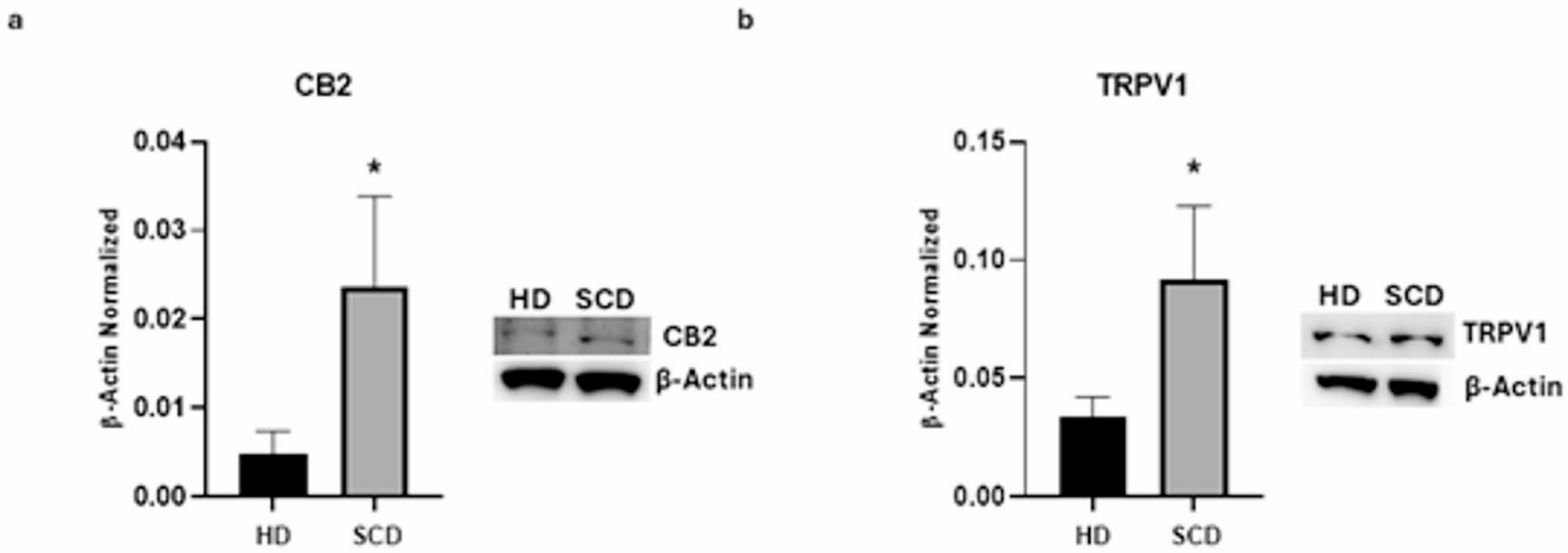
CB2 and TRPV1 expression in SCD macrophages obtained from SCD pediatric/young patients. CB2 (**a**), TRPV1 (**b**) protein expression levels in sickle cell disease (SCD) macrophages compared to those of healthy donors (HD), evaluated by Western blotting starting from 15 µg of total lysates. The most representative images are displayed. The protein bands were detected through Image Lab Ink 6.1 software “BIORAD”, and the intensity ratios of immunoblots compared to HD, taken as 1, were quantified after normalizing with the respective controls. The relative quantification for these proteins, normalized for the housekeeping protein β-actin, is represented in the histograms as the mean ± SD. An unpaired t-test was performed for statistical analysis. *, *p* ≤ 0.05 compared to HD. Original blots/gels are presented in Supplementary Uncropped WB Figures.

To evaluate CB2 and TRPV1 protein expression levels in SCD macrophages, we performed several western blots. Biochemical analysis showed that, in SCD macrophages, CB2 was remarkably higher than HD macrophages (*p* = 0,0376) with a very large effect size (η^2^ = 0,7009; 95% CI [0,001742; 0,03564]) (Fig. 3A), thus suggesting that the altered expression of this receptor could be involved in the impaired inflammatory state and in pain development in SCD patients. We also observed a statistically significant increase of TRPV1 protein expression levels in SCD macrophages compared to HD ones (*p* = 0,0362) with a very large effect size (η^2^ = 0,7062; 95% CI [0,006066; 0,1099] (Fig. 3B). This result confirmed the already known involvement of TRPV1 in inflammation and in SCD associated pain, suggesting for the first time the involvement of this receptor in human SCD associated pain.

### Effects of CB2 and TRPV1 stimulation on SCD macrophages polarization

We evaluated the effects of JWH-133, AM630, RTX and I-RTX on macrophage polarization markers by performing Western blotting.

**Fig. 4:**
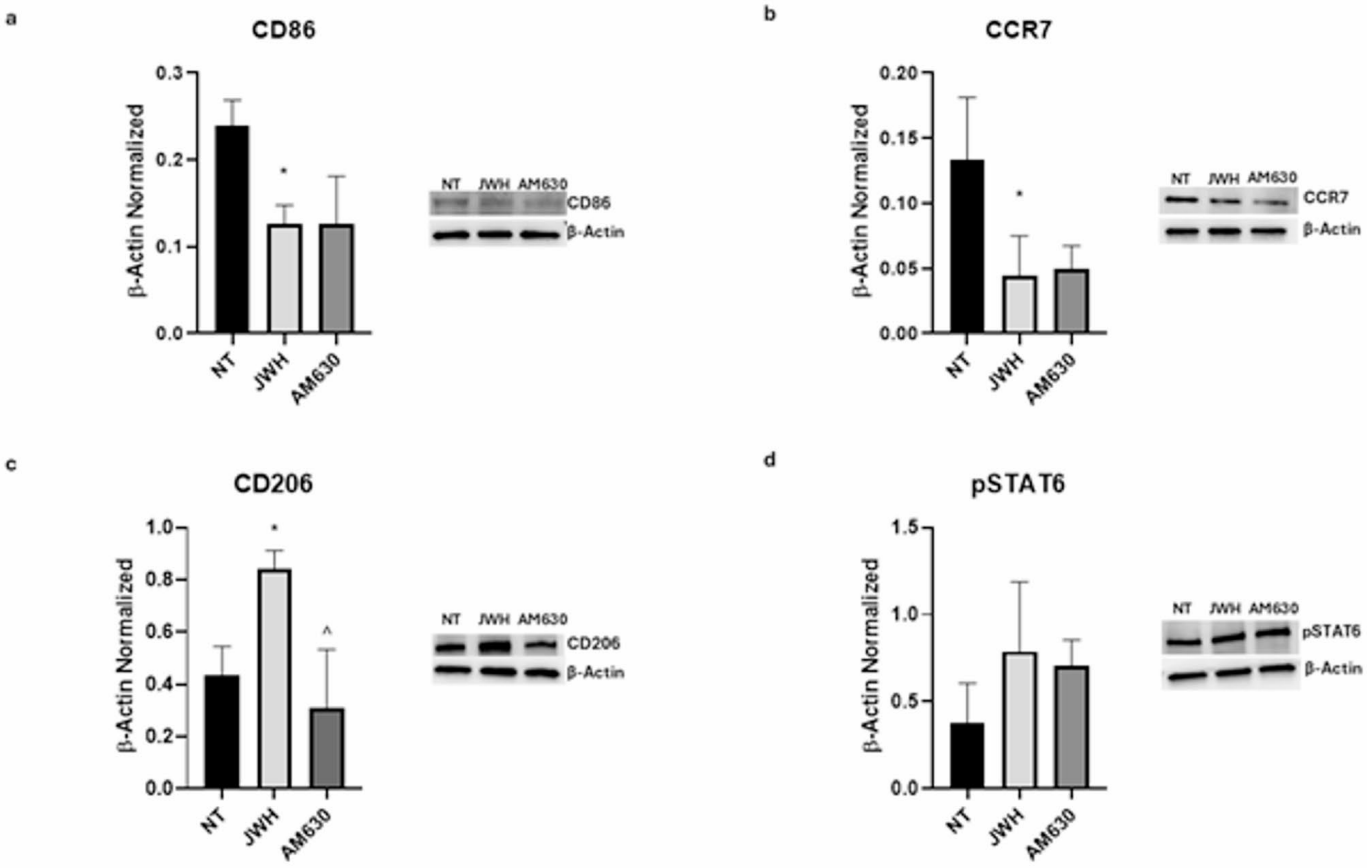
Effects of CB2 stimulation on macrophages derived from SCD pediatric/young patients. CD86 (**a**), CCR7 (**b**), CD206 (**c**), and pSTAT6 (**d**) protein expression levels sickle cell disease (SCD) macrophages after 24 h treatment with JWH-133 and AM630 evaluated by Western blotting, starting from 15 µg of total lysates. The most representative images are displayed. The protein bands were detected through Image Lab Ink 6.1 software “BIORAD”, and the intensity ratios of immunoblots compared to non-treated (NT) macrophages, taken as 1, were quantified after normalizing with the respective controls. The relative quantification for these proteins, normalized for the housekeeping protein β-actin, is represented in the histograms as the mean ± SD. For statistical analysis, a Shapiro-Wilk Normality test was used to asses whether the distribution of our samples were normal or not. For samples with normal distribution (**a**, **c**, **d**), we performed one way ANOVA test followed by Tukey HSD as post hoc. For samples without a normal distribution (**b**), we employed a Kruskal-Wallis Test, followed by the Dunn’s Test as post hoc. *, *p* ≤ 0.05 compared to NT; ^, *p* ≤ 0.05 compared to JWH-133. Original blots/gels are presented in Supplementary Uncropped WB Figures.

Treatment with the CB2 selective agonist, JWH-133, induced a reduction of both M1 markers, CD86 and CCR7, in a statistically manner in SCD treated macrophages compared to untreated (NT) ones (*p* = 0,0156 and *p* = 0,0436, respectively) (Fig. 4A, B). This effect was supported by a moderate but relevant effect size (R² = 0,6806) and a 95% confidence interval ([0,003152; 0,1751]) that does not include zero, indicating a statistically and potentially biologically relevant reduction. On the other side, after CB2 stimulation, we observed a statistically significant increase of M2 marker CD206 (Fig. 4C), and a trend to increase of pSTAT6, even though not in a statistically manner (Fig. 4D), compared to the NT macrophages (*p* = 0,0368 and *p* = 0,2465, respectively). The effect size was high for CD206 (R² = 0,7737) and moderate for pSTAT6 (R² = 0,3775). The 95% confidence interval for CD206 ([−0,7831; −0,03086]) does not include zero, confirming the statistical significance and biological relevance of the observed increase. In contrast, the confidence interval for pSTAT6 ([−1,113; 0,2888]) includes zero, indicating that the observed trend is not conclusive but could be affected by the limited sample size. Overall, these findings suggest that CB2 activation promotes an M2-like phenotype, with a clear upregulation of CD206 and a potential involvement of the STAT6 pathway that warrants further investigation.

Instead, AM630 treatment induced an opposite effect or no appreciable change compared to JWH-133 treatment (Fig. 4). Specifically, no relevant variations were observed in the expression levels of CD86, CCR7, and pSTAT6 following AM630 administration (*p* > 0,9999, *p* = 0,9830 and *p* = 0,9299, respectively). These findings were further supported by the effect size, which was moderate for CCR7 and pSTAT6 (R^2^ = 0,6806 and R^2^ = 0,3775, respectively) and by 95% CI ([−0,09092; 0,08103] for CCR7 and [−0,6175; 0,7844] for pSTAT6), both of which include zero, indicating a lack of statistically meaningful effects. Regarding CD206, AM630 treatment induced an opposite effect by significantly reducing CD206 levels compared to JWH-133 treatment (*p* = 0,0117). This was supported by a high effect size (R² = 0,7737) and a 95% CI ([0,1545; 0,9067]) that does not include zero, further validating the significance of the result. Altogether, these findings suggest that CB2 blockade does not significantly alter the expression of CD86, CCR7, and pSTAT6, but induces an opposite effect on CD206 compared to JWH-133 administration, reinforcing the specific role of CB2 activation in modulating macrophage polarization.

**Fig. 5:**
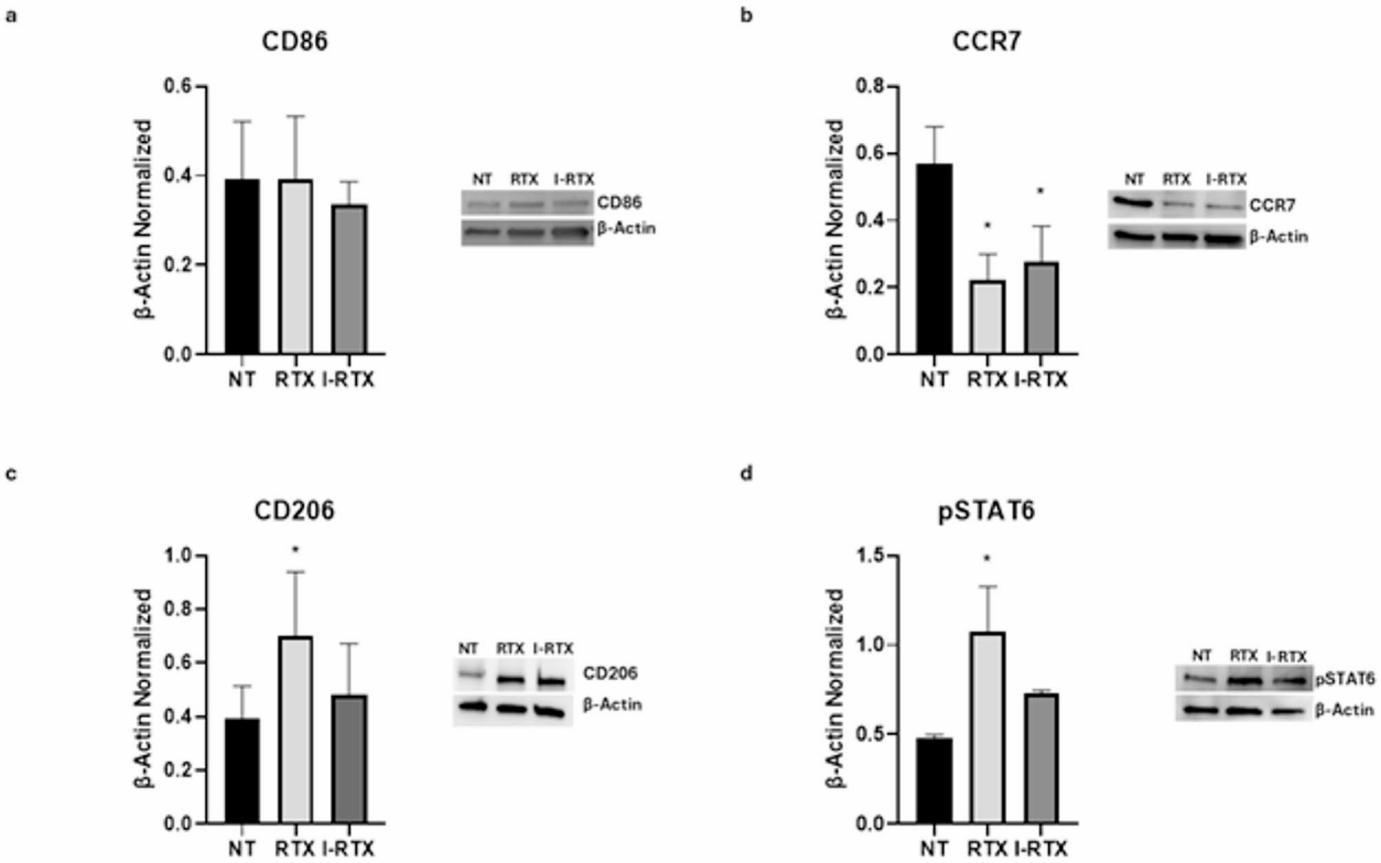
Effects of TRPV1 stimulation on macrophages derived from SCD pediatric/young patients. CD86 (**a**), CCR7 (**b**), CD206 (**c**), and pSTAT6 (**d**) protein expression levels sickle cell disease macrophages after 24 h treatment with RTX and I-RTX evaluated by Western blotting, starting from 15 µg of total lysates. The most representative images are displayed. The protein bands were detected through Image Lab Ink 6.1 software “BIORAD”, and the intensity ratios of immunoblots compared to non-treated (NT) macrophages, taken as 1, were quantified after normalizing with the respective controls. The relative quantification for these proteins, normalized for the housekeeping protein β-actin, is represented in the histograms as the mean ± SD. For statistical analysis, a Shapiro-Wilk Normality test was used to asses whether the distribution of our samples were normal or not. All samples showed normal distribution, and we performed one way ANOVA test followed by Tukey HSD as post hoc. *, *p* ≤ 0.05 compared to NT. Original blots/gels are presented in Supplementary Uncropped WB Figures.

Stimulation of TRPV1 receptor with RTX determined a strong reduction of protein expression levels of M1 marker CCR7 (*p* = 0,0127) (Fig. 5B), and a marked increase of both CD206 (*p* = 0,0315) and pSTAT6 (*p* = 0,0060) protein expression levels (Fig. 5C, D). The high effect size for CCR7 (R^2^ = 0,7782) and pSTAT6 (R^2^ = 0,8059), and the moderate effect size of CD206 (R^2^ = 0,3630), together with the 95% CI ([0,09639; 0,5951], [−0,5905; −0,02624], [−0,9604; −0,2273], respectively) not including zero, confirmed the biological relevance and robustness of the observed effects induced by RTX. However, RTX did not change the protein expression levels of CD86 (*p* = 0,9998) (Fig. 5A). This result was supported by the low effect size (R^2^ = 0,07618) and the 95% CI ([−0,2840; 0,2876]) which includes zero, thus confirming the absence of a statistically significant effect.

I-RTX, similarly to AM630, did not induce any significant variation, but rather showed a trend toward a reverse effect in the expression levels of the analyzed proteins compared to RTX treatment (Fig. 5). In particular, I-RTX treatment was associated with a non-significant trend toward increased CCR7 levels compared to RTX (*p* = 0,8045). Additionally, we observed a trend toward decreased expression of both CD206 and pSTAT6 following I-RTX treatment compared to RTX, although not statistically significant (*p* = 0,1407 and *p* = 0,0637, respectively). These trends were supported by a high effect size for CCR7 (R² = 0,7782) and pSTAT6 (R² = 0,8059), and a lower effect size for CD206 (R² = 0,3630). The corresponding 95% CI ([−0,3014; 0,1973] for CCR7, [−0,06234; 0,5020] for CD206, and [−0,02324; 0,7099] for pSTAT6) all included zero, suggesting that while the observed differences were not statistically significant, they may indicate a potential inverse modulation that warrants further investigation in studies with increased sample size. Regarding CD86 no statistically significant variation was observed after I-RTX treatment compared to RTX (*p* = 0,8256) This result was further supported by the low effect size (R^2^ = 0,07618) and the 95% CI ([−0,2300; 0,3417]), which includes the zero, thereby confirming the lack of a statistically significant effect.

### Effects of CB2 and TRPV1 stimulation on inflammatory state in SCD macrophages

To evaluate the effect of CB2 and TRPV1 stimulation on inflammatory state in SCD macrophages, we performed several ELISA.

Both JHW-133 and RTX exhibit anti-inflammatory effects by determining a strong reduction of IL-6 and TNF-α pro-inflammatory cytokines levels, which appeared to be statistically significant only for IL-6 after JWH-133 administration (Supplementary Figure S2 A, B; Supplementary Figure S3 A, B). We observed a highly significant reduction in IL-6 levels upon JWH treatment (*p* < 0.0001), with a very high effect size (R² = 0.9784; 95% CI [5.219; 8.479]). TNFα expression was not significantly altered by either JWH or RTX treatments. For JWH, the decrease was not statistically significant (*p* = 0.2412), although the effect size was moderate (R² = 0.3568; 95% CI [−3,651; 14,32]), suggesting that approximately 36% of the variation could be explained by the treatment. For RTX, while the observed reduction was not significant (*p* = 0.1616), the effect size was relatively high (R² = 0.5246; 95% CI [−2,463; 13,82]), indicating that over half of the variance is related to treatment. Meanwhile, the inverse agonist AM630 and the antagonist I-RTX caused a reduction of both these two inflammatory cytokines compared to NT. However, we noticed no statistically significant difference between AM630 and JWH effects on IL-6 and TNF-α (*p* = 0,1124 and *p* = 0,6040, respectively), but there is a noticeable trend. Moreover, the R² (0,9784 and 0,3568, respectively) suggests a moderate relationship between the two treatments. The 95% CI ([−0,321; 2,918] and [−11,91; 6,060], respectively) includes zero, but the positive trend observed could suggest a potential effect that might become significant with larger sample sizes. We did not observe statistically significant differences between I-RTX and RTX effects on IL-6 and TNF-α (*p* > 0,9999 and *p* = 0.9848, respectively). The moderate R² value (0,5246) for TNF-α suggests that there might be a pattern emerging. The 95% CI [−7,699; 8,584] for TNF-α includes zero, but the slight trend suggests that further studies could reveal more definitive effects.

Moreover, CB2 stimulation also induced a trend to increase of the anti-inflammatory cytokines IL-10 not statistically significant (*p* = 0,7276), but the analysis revealed a moderate effect size (R²=0,4766, 95% CI [−48,90; 29,06]), suggesting a possible treatment effect. The stimulation of TRPV1 determined a trend to reduction of IL-10, even though not in statistically significant manner, revealing a lack of significant differences among groups (*p* = 0.7957), with a very low effect size (R²=0.0772; 95% CI [−35,62; 55,02]) (Supplementary Figure S2 C; Supplementary Figures S3 C).

; IL-4 levels did not show significant changes following either JWH or RTX treatment. JWH-133 induced no significant variation between groups (*p* = 0.9876), and the effect size was negligible (R² = 0.0042; 95% CI [−5,898; 5,438]), indicating almost no variance explained by treatment. Also RTX induced non-significant variation in IL-4 levels (*p* = 0.5146), with a low effect size (R² = 0.1986; 95% CI [−6,893; 3,398]), suggesting that treatment explains less than 20% of the observed variance Supplementary Figure S2 D; Supplementary Figures S3 D).

Finally, considering the effect of AM630 and I-RTX on anti-inflammatory cytokines levels, we noticed an opposite pharmacological behavior compared to those ones induced by the selective agonists (Supplementary Figure S2; Supplementary Figure S3). Nevertheless, the differences in IL-4 and IL-10 levels observed following AM630 and JWH administration did not reach statistical significance (*p* = 0,9882 and *p* = 0,1320, respectively). However, a clear trend can be noted, particularly for IL-10. The R² values (0,0042 for IL-4 and 0,4766 for IL-10) suggest a negligible association in the first case and a moderate relationship in the second. Additionally, the 95% confidence intervals [−5,397; 5,940] for IL-4 and [−9,779; 68,18] for IL-10 both include zero, reinforcing the lack of statistical significance.

Nonetheless, these findings, particularly the increase in IL-10 levels, could be affected by the relatively small sample size hiding a potential an opposite regulatory effect of the two treatments on this cytokine. No statistically significant differences were observed between the effects of I-RTX and RTX on IL-4 and IL-10 levels (*p* = 0,5649 and *p* = 0,8460, respectively). The R² values (0,1986 for IL-4 and 0,0771 for IL-10) indicate a weak to moderate association, which may point to an emerging pattern. Although the 95% confidence intervals ([−3,352; 6,939] for IL-4 and [−53,57; 37,07] for IL-10) include zero, suggesting non-significance, the slight trends observed hint at potential differences that could become clearer with increased statistical power.

### Effects of CB2 and TRPV1 stimulation on iron metabolism in SCD macrophages

We performed several Western Blot and Iron Assays to evaluate the effect of CB2 and TRPV1 stimulation on iron metabolism markers in SCD macrophages.

**Fig. 6:**
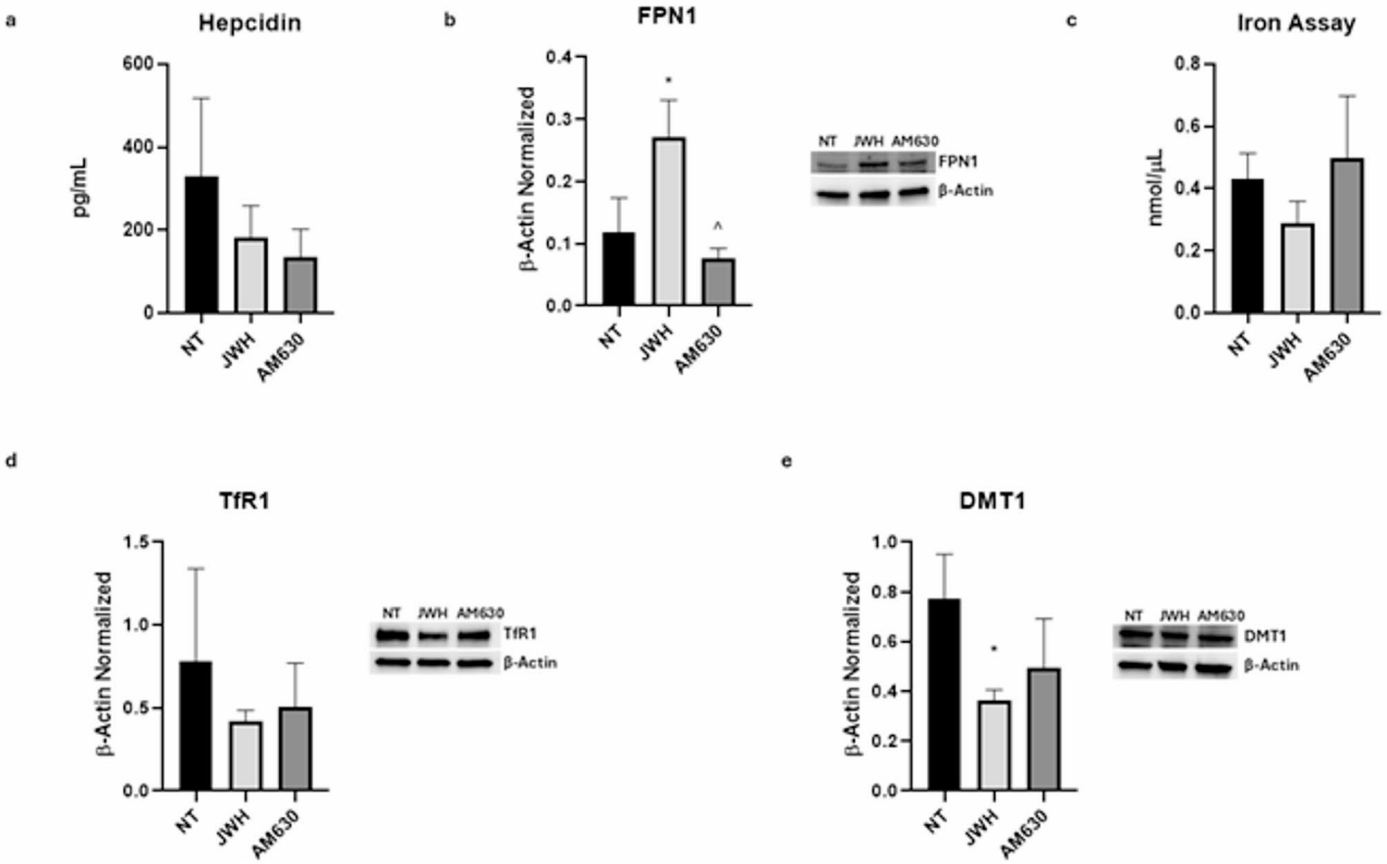
Effects of CB2 stimulation on iron metabolism modulation in macrophages derived from SCD pediatric/young patients. Evaluation of hepcidin (**a**) release from sickle cell disease (SCD) macrophages compared to healthy donors’ (HD) macrophages, revealed through an enzyme-linked immunosorbent assay (ELISA). The graphs show hepcidin levels (pg/mL) as the mean ± standard deviation (SD). For statistical analysis, a Shapiro-Wilk Normality test was used to asses whether the distribution of our samples were normal or not. The samples showed normal distribution, and we performed one way ANOVA test followed by Tukey HSD as post hoc. FPN1 (**b**), TfR1 (**d**), and DMT1 (**e**) protein expression levels in SCD macrophages of pediatric/young patients’ compared to NT, evaluated by Western blotting, starting from 15 µg of total lysates. The most representative images are displayed. The protein bands were detected through Image Lab Ink 6.1 software “BIORAD”, the intensity ratios of immunoblots compared to NT, taken as 1, were quantified after normalizing with the respective controls. The relative quantification for these proteins, normalized for the housekeeping protein β-Actin, is represented in the histograms as the mean ± SD. For statistical analysis, a Shapiro-Wilk Normality test was used to asses whether the distribution of our samples were normal or not. All samples showed normal distribution, and we performed one way ANOVA test followed by Tukey HSD as post hoc. *, p ≤ 0.05 compared to NT; ^, p ≤ 0.05 compared to JWH-133. Original blots/gels are presented in Supplementary Uncropped WB Figures. Fe3+ (**c**) intracellular concentrations (nmol/µL) in NT and SCD macrophages of pediatric/young patients’, determined by iron assay. Histogram shows Fe3 + concentration as the mean ± SD. For statistical analysis, a Shapiro-Wilk Normality test was used to asses whether the distribution of our samples were normal or not. The samples showed normal distribution, and we performed one way ANOVA test followed by Tukey HSD as post hoc.

**Fig. 7:**
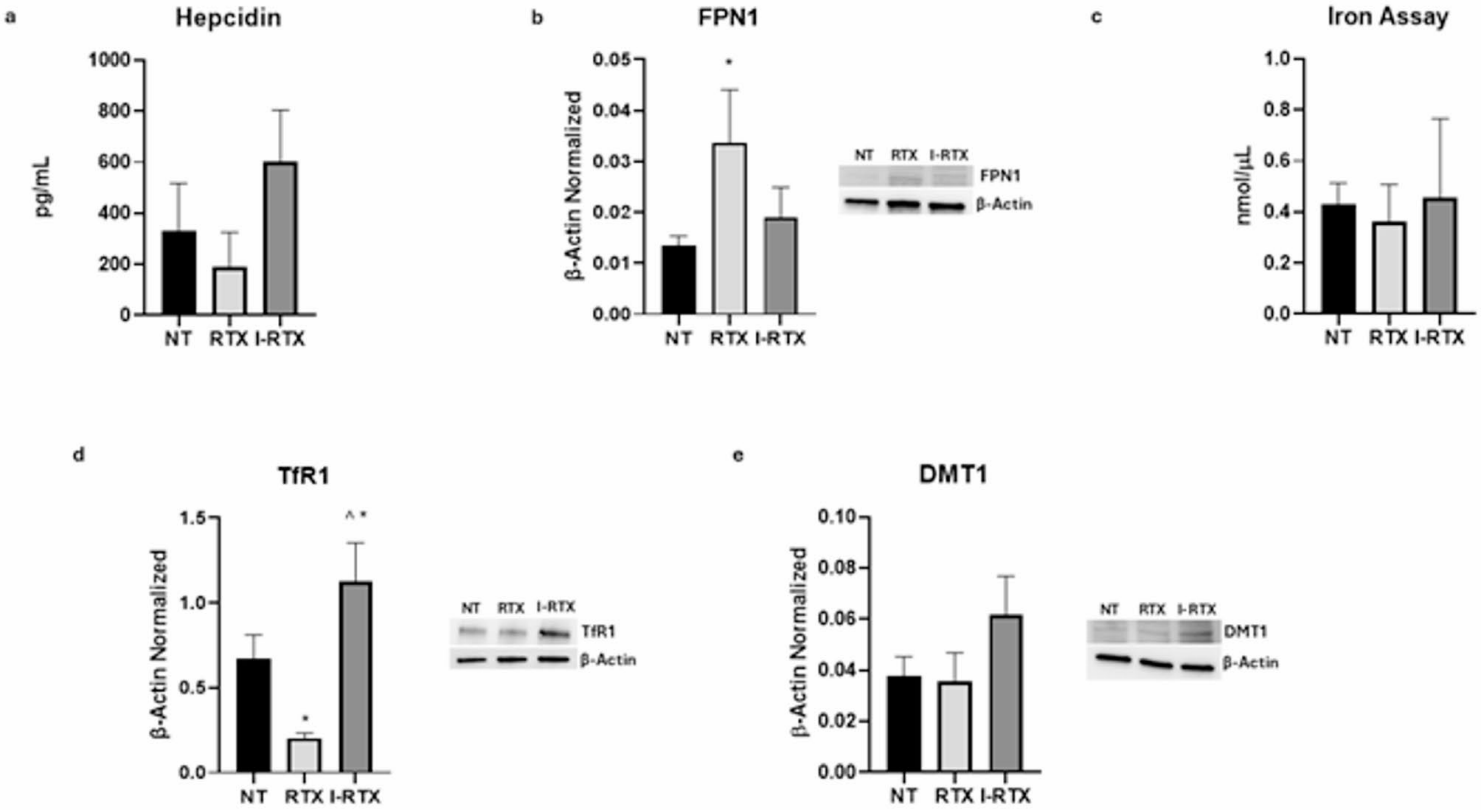
Effects of TRPV1 stimulation on iron metabolism modulation in macrophages derived from SCD pediatric/young patients. Evaluation of hepcidin (**a**) release from sickle cell disease macrophages compared to healthy donors’ (HD) macrophages, revealed through an enzyme-linked immunosorbent assay (ELISA). The graphs show hepcidin levels (pg/mL) as the mean ± standard deviation (SD). For statistical analysis, a Shapiro-Wilk Normality test was used to asses whether the distribution of our samples were normal or not. The samples showed normal distribution, and we performed one way ANOVA test followed by Tukey HSD as post hoc. FPN1 (**b**), TfR1 (**d**), and DMT1 (**e**) protein expression levels in SCD macrophages of pediatric/young patients’ compared to NT, evaluated by Western blotting, starting from 15 µg of total lysates. The most representative images are displayed. The protein bands were detected through Image Lab Ink 6.1 software “BIORAD”, the intensity ratios of immunoblots compared to NT, taken as 1, were quantified after normalizing with the respective controls. The relative quantification for these proteins, normalized for the housekeeping protein β-Actin, is represented in the histograms as the mean ± SD. For statistical analysis, a Shapiro-Wilk Normality test was used to asses whether the distribution of our samples were normal or not. All samples showed normal distribution, and we performed one way ANOVA test followed by Tukey HSD as post hoc. *, p ≤ 0.05 compared to NT; ^, p ≤ 0.05 compared to JWH-133. Original blots/gels are presented in Supplementary Uncropped WB Figures. Fe3+ (**c**) intracellular concentrations (nmol/µL) in NT and SCD macrophages of pediatric/young patients’, determined by iron assay. Histogram shows Fe3 + concentration as the mean ± SD. For statistical analysis, a Shapiro-Wilk Normality test was used to asses whether the distribution of our samples were normal or not. No samples showed a normal distribution and we employed a Kruskal-Wallis Test, followed by the Dunn’s Test as post hoc.

Firstly, we evaluated the hepcidin-FPN-1 axis. We showed that both JWH-133 and RTX induced a trend toward reduction of Hepcidin release, even though not in a statistically manner although not statistically significant (*p* = 0,3633 and *p* = 0,6196, respectively), with a moderate to high effect size (R² = 0,4083 and R² = 0,5852, respectively; 95% CI [–158,9; 455,6] and [–302,3; 582,3], respectively).This observation correlates with the strong statistically increase of the protein expression levels of the only known iron exporter, FPN-1, after the administration of JWH-133 and RTX (*p* = 0,0183 and *p* = 0,0271, respectively) (Figs. 6A and B and 7A and B). This result is further supported by the high effect size (R^2^ = 0,8202 for JWH-133 and R^2^ = 0,6950 for RTX) and by 95% CI ([−0,2742; −0,03341]; [−0,03770; −0,002895]) that do not include zero, confirming the robustness and potential biological relevance of the observed upregulation. We also assessed the protein expression levels of the two iron importers, TfR-1 and DMT-1, showing that, JHW-133 induced a statistically significant reduction in DMT1 expression levels (*p* = 0,0420) as well as a trend to decrease of TfR1which was not statistically significant (*p* = 0,4893). These results were supported by a moderate but relevant effect size for DMT1 (R² = 0,6413) and a lower effect size for TfR1 (R² = 0,2113). The 95% CI for DMT1 ([0,01811; 0,7965]) does not include zero, suggesting a potentially meaningful effect, while the interval for TfR1 ([−0,5457; 1,258]) includes zero. Nevertheless, the observed trend for TfR1 may warrant further investigation, as increasing the sample size could help confirm this effect. We revealed that also RTX determined a strong reduction of TfR-1 (*p* = 0,0245), while no change was observed for DMT-1 (*p* = 0,9827) (Fig. 7D, E). Effectively, these results were supported by a high effect size for TfR1 (R²=0,8966) and a moderate effect size for DMT1 (R²=0,6061). The 95% CI for TfR1[0,07594; 0,8593] did not include zero, confirming the statistical and biological relevance of the effect. Conversely, the 95% CI for DMT-1 [−0,02753; 0,03092], ([−0.02753; 0.03092]) included zero, indicating the absence of a statistically significant change. Moreover, after both CB2 and TRPV1 stimulations, we highlighted a trend to reduction of intracellular iron concentration, even though not in statistically significant manner both after JWH-133 and RTX treatments (*p* = 0,4372 and *p* > 0,9999, respectively), with a moderate effect size (R^2^ = 0,3905) and 95% CI ([−0,1880; 0,4706]) includes zero, suggesting a potential trend that could be clarified with a larger sample size. These results, all together, let us speculate that CB2 and TRPV1 stimulations could modulate iron metabolism by acting on both hepcidin-FPN1 axis and on the protein expression levels of its cell importers, causing a reduction of its internalization thus limiting cell damage and inflammation related to iron metabolism dysregulation.

Regarding AM630 and I-RTX, both drugs exhibited a reverse effect compared to the agonists (Figs. 6 and 7), validating our findings. In particular, we observed a statistically significant reduction of FPN-1 protein expression level after AM630 administration compared to JWH-133 treatment (*p* = 0,0061). This result was supported by a very high effect size (R² = 0,8202) and by the 95% CI ([0,07427; 0,3151]) which does not include zero, suggesting a robust and biologically relevant downregulation of FPN-1 following CB2 receptor blockade. Regarding DMT1 and TfR1, we observed a trend toward increased expression following AM630 treatment compared to the effects induced by JWH-133 (*p* = 0,5781 and *p* = 0,9571, respectively). A moderate but relevant effect size was found for DMT1 (R² = 0,6413), while a lower effect size was observed for TfR1 (R² = 0,2113). However, the 95% confidence intervals for both DMT1 ([−0,5218; 0,2566]) and TfR1 ([−0,9852; 0,8188]) include zero, suggesting that these differences are not statistically meaningful. However, the observed trends may warrant further investigation in studies with larger sample sizes to better clarify and potentially confirm these effects. We also did not observe any relevant variation in hepcidin levels following AM630 treatment compared to JWH-133 (*p* = 0,8886), with a low effect size (R² = 0,4083) and a 95% confidence interval ([−260,4; 354,1]) that includes zero. Therefore, these results suggest no significant modulation of hepcidin expression under these conditions, although further studies may be needed to confirm this finding. Finally, we noticed a trend to increase of intracellular iron concentration after AM630 administration compared to JWH-133 treatment, but not in statistically significant manner (*p* = 0,2143). The observed low effect size (R² = 0,3905) and 95% CI ([−0,5350; 0,1236]) including zero, reveal no significant modulation.

Considering I-RTX effects compared to RTX, we observed a reduction in FPN-1 protein expression levels that did not reach statistical significance (*p* = 0,0903). This finding was accompanied by a moderate effect size (R² = 0,6950) and a 95% confidence interval ([−0,002678; 0,03213]) including zero, suggesting a possible trend that warrants further investigation with larger sample sizes to clarify the biological relevance. I-RTX administration also induced a statistically significant increase in TfR1 protein expression levels compared to RTX treatment (*p* = 0,0009). This result was supported by a high effect size (R² = 0,8966) and a 95% confidence interval ([−1,312; −0,5289]) that does not include zero, further confirming the robustness and biological relevance of this statistically significant finding. Moreover, we observed a non-statistically significant increase in DMT1 protein expression following I-RTX administration compared to RTX treatment effects (*p* = 0,0776), supported by a moderate effect size (R² = 0.6061) and a 95% CI ([−0.05509; 0.003357]) including zero, thus suggesting a trend that may warrant further investigation with an increased sample size. A trend toward increased levels was also observed for hepcidin and intracellular iron concentration following I-RTX administration compared to RTX treatment, although these changes were not statistically significant (*p* = 0,0647 and *p* > 0.9999, respectively). The moderate effect size for hepcidin (R² = 0,5852), together with the 95% CI including zero ([−854,6; 29,94]), confirms the non-statistically significant result and indicates that further studies with larger sample sizes may be required to clarify this trend.

## Discussion

SCD is a multisystem monogenic disorder affecting almost every organ in the body^5,50,51^ and contributes significantly to morbidity and premature mortality in pediatric patients^1,52,53^. Polymerization of HbS (βGlu6Val) causes sickling of RBC which determines persistent inflammatory state and vasculopathy^54,55^; particularly, the rigidity and the frangibility of sickled RBCs are responsible for anemic condition and VOC, which trigger leukocytes, endothelial cells, and platelets, and heighten inflammation^3,4^. Systemic inflammation, repeated vascular occlusion, and inflammation, lead SCD patients to also experience acute pain and chronic daily pain^22–24^, which include neuropathic, inflammatory, and nociceptive mechanisms^23^. Actually, several drugs are use in order to manage inflammation and pain, even though pain is not completely resolved with them.

Therefore, the discovery of novel therapeutic approach and target to counteract inflammation and manage pain in SCD could be useful.

Our study aimed to better understand the role of macrophages in SCD inflammation, and to propose novel possible therapeutic targets to counteract the severe consequences induced by inflammatory state impairment in SCD patients.

Firstly, we investigated macrophages phenotype and we studied the iron metabolism involvement in the inflammatory processes.

This manuscript shows for the first time to our knowledge the assessment of pediatric/young adults SCD macrophages phenotypes, revealing high expression levels of M1 macrophages phenotypic markers, CCR7 and CD86, together with remarkable lower expression levels of the M2 phenotype marker, CD206, in SCD macrophages compared to the HD ones. We also evaluated pSTAT6 protein expression levels, a protein involved in macrophages phenotype switch from M1 pro-inflammatory type to M2 anti-inflammatory one. Interestingly, we observed a lower protein expression level of pSTAT6 in SCD macrophages compared to HD. Taken together, these data suggest a prevalence of M1 pro-inflammatory macrophages in SCD pediatric/young adults patients, which could be involved in the impaired inflammatory state of these patients.

To better characterize the inflammatory state in SCD macrophages, we also studied the inflammatory cytokines released by these cells. We observed a strong increase of both pro-inflammatory cytokines levels, IL-6 and TNF-α, and also a trend to reduction of the anti-inflammatory cytokine IL-10. These results confirm the presence of an impairment of the inflammatory state in SCD macrophages, which is a typical condition of this disease.

We then moved forward studying iron homeostasis in SCD macrophages to investigate its possible alteration, which could be strongly related to the impaired inflammatory state of SCD patients. It is indeed known that iron metabolism and inflammation are two tightly associated processes. As expected, we found that the high levels of SCD-derived macrophages IL-6 were associated with a strong increase of hepcidin release by SCD macrophages compared to HD ones. Hepcidin is an antimicrobial peptide, produced mostly either by hepatocytes or macrophages, which also negatively regulates iron in circulation by controlling iron absorption from dietary sources and iron release from macrophages^14^; as a consequence of increased levels of hepcidin, we also detected a reduction of the protein expression levels of the only known iron exporter, FPN-1. Interestingly, we demonstrated that SCD macrophages showed high expression levels of both iron importers, DMT1 and TfR1, compared to HD. These results suggest the presence of an impaired iron metabolism in SCD macrophages, which could be involved in an impaired systemic inflammation response (prevalence of M1 macrophages) as well as be linked to the SCD-chronic anemia. Indeed, the contribution of hepcidin as a regulator of iron metabolism and erythropoiesis on the severity of anemia in SCD remains poorly characterized^56^ and its circulating levels seem to not significantly relate to any clinical or laboratory parameters^57^. Our findings could suggest a linkage among systemic unbalance of macrophagic polarization, local restriction in iron availability, inflammation and anemia.

Moreover, as previously mentioned, excessive inflammation contributes to the development and degree of pain. Our research group already described the important role of macrophage polarity to counteract inflammation state along with iron load in other disease^15,18–20^, therefore with this study we propose macrophages phenotype and iron metabolism components as novel therapeutic targets in SCD to counteract inflammation, also potentially offering insights into pain development and perception associated with the dysregulated inflammatory state in SCD.

Moreover, considering the importance to identify novel therapeutic approaches to contain inflammation in these patients, and considering the involvement of CB2 and TRPV1 in both inflammation and pain modulation, we decided to investigate the expression of these receptors in SCD macrophages and the effects of their stimulation on inflammation in SCD macrophages.

Interestingly, we revealed high protein expression levels of both CB2 and TRPV1, thus confirming a plausible role exerted by these receptors in inflammation and pain in SCD. It is known that macrophages also have a key role in pain by communicating with nociceptors, activating them through the release of pro-inflammatory molecules and responding to their stimuli^58^. Therefore, macrophages could be considered not only a target to counteract inflammation but also to manage pain in SCD patients. Although several studies have examined the role of CB2 and TRPV1 in SCD, none have specifically investigated their function and, in particular, the effects of their stimulation in macrophages isolated from the peripheral blood of SCD patients. Following this conceptual framework, we aimed to evaluate the effects of CB2 and TRPV1 stimulation with their selective agonists, JWH-133 and RTX respectively, on macrophages isolated from SCD pediatric and young adults patients, assessing their effects after 24 h of incubation. Initially, we verified their anti-inflammatory activity by observing reductions of two M1 phenotype markers, CCR7 and CD86, along with concurrent increases in M2 phenotype markers, CD206 and pSTAT6. These findings confirmed the well-documented ability of CB2 and TRPV1 stimulation in inducing anti-inflammatory effects and, for the first time to our knowledge, we demonstrated that their stimulation could promote a macrophage switch towards the anti-inflammatory phenotype in SCD. This result represents a significant finding, as it highlights a novel property of these receptors in modulating inflammation through the regulation of macrophage phenotype in SCD. Additionally, we corroborated the anti-inflammatory effects of these compounds by observing a remarkable decrease in pro-inflammatory cytokines IL-6 and TNF-α, coupled with a trend toward increased levels of the anti-inflammatory cytokines IL-10.

We further highlighted the effects of the CB2 and TRPV1 selective agonists – JWH-133 and RTX, respectively – on iron metabolism. Unexpectedly, we found that administering these two drugs induced changes in markers of iron metabolism by reducing intracellular iron concentrations, even though not in statistically significant levels. Specifically, CB2 and TRPV1 stimulation not only resulted in a marked reduction in IL-6 release, but also led to a decrease in hepcidin levels and an increase in FPN-1 protein expression. Moreover, interestingly, both drugs also determined a strong reduction of protein expression levels of both iron importer, TfR1 and DMT1, thus contributing to reduce iron internalization. The iron metabolism components modulation induced by CB2 and TRPV1 stimulation could be responsible for ameliorating the impaired inflammatory state in SCD.

Moreover, to demonstrate that the observed effects following the administration of the selective agonists were specifically due to CB2 and TRPV1 receptors stimulation, we also performed treatments using the CB2 inverse agonist, AM630, and the TRPV1 antagonist, I-RTX. We showed that the administration of AM630, and particularly of I-RTX, seems to induce a trend toward a reversal of the effects of JWH and RTX both in terms of macrophage phenotype and iron metabolism, even though the current analysis did not reveal statistically significant results. This trend, although not yet reaching significance, suggests that with a larger sample size and further testing, we may observe more definitive reversal effects.

Our results reveal an alteration of macrophages polarity and of iron metabolism, suggesting us that targeting macrophages and iron could be a novel interesting therapeutic approach to tackle iron burden and inflammation, which are characteristic in SCD. Moreover, the observed increase of CB2 and TRPV1 protein expression levels let us suppose that these receptors could also be interesting remarkable targets to suppress the altered inflammatory state and potentially to counteract inflammatory-related pain in SCD patients.

Therefore, although some findings were not statistically significant, our results, for the first time, highlighted that CB2 and TRPV1 stimulation exerted direct anti-inflammatory effects, by modulating both the release of inflammatory cytokines and macrophage polarization, and an indirect anti-inflammatory property, by determining changes in components involved in iron metabolism in SCD macrophages. Our findings are of particular relevance, as they unveil a previously unrecognized role of these receptors in attenuating inflammation by modulating macrophage polarization and determining changes in expression levels and in concentration of several components involved in iron metabolism regulation in SCD macrophages.

Moreover, our data suggest that an unbalanced forced macrophagic polarization in SCD could be preferentially targeted by usage of CB2 and TRPV1 agonists that would exert immune-modulating effects beneficial on several iron and not-iron related features (i.e. anemia and pain), as already suggested by animal models^30–32^.

Certainly, our study serves as an initial step in exploring biological mechanisms driving inflammation and pain in SCD patients, highlighting the roles of TRPV1 and CB2 receptors. Even though this study presents some limitations, including the exclusive use of an in vitro model and the lack of functional assessments of pain or in vivo validation, it nonetheless provides new consistent and relevant insights. Although the number of subjects may appear limited, it is appropriate for a preclinical in vitro study involving primary macrophages isolated from the peripheral blood of pediatric SCD patients, considering the inherent challenges of obtaining and working with primary human cells, particularly from pediatric cohorts.

Concluding, our results provide novel and clear evidence demonstrating expression of CB2 and TRPV1 receptors in macrophages derived from the peripheral blood of pediatric patients with SCD, a finding not previously reported in patients affected by this disease. Biologically, our data suggest that CB2 and TRPV1 may contribute to modulate inflammatory responses in SCD, at least in part through the regulation of macrophage phenotype and iron metabolism-related pathways. This dual role in both immune regulation and iron homeostasis management highlights a potential intersection between chronic inflammation and dysregulated iron homeostasis, which are hallmark features of SCD pathophysiology. In terms of clinical translational potential, these findings indicate CB2 and TRPV1 as promising molecular targets for future therapeutic strategies to reduce inflammation and its downstream complications in SCD. Further studies will be essential to validate these targets in vivo and to explore their relevance in clinical practice, including their potential impact on pain and organ damage associated with the disease.

Importantly, our data introduce original and compelling evidence for a role of CB2 and TRPV1 receptors in counteracting inflammation, potentially through the modulation of iron metabolism pathways. These findings highlight new therapeutic perspectives in SCD. However, further in vitro and in vivo studies will be necessary to better understand the mechanisms of action of these receptors and to validate the causality of the observed effects within a more complex physiological context. In particular, incorporating functional pain assessments and in vivo models will be crucial to fully explore the translational implications of targeting these pathways in SCD.

## Materials and methods

### Source of macrophages

Macrophages were obtained from 32 partecipants in total, among them 16 were SCD patients, indeed homozygous for c.17 A > C; p.Glu6Val (median age 9 years old) (Supplementary Table S1) and the other 16 were healthy donors (HD). All participants were enrolled at the Department of Women, Child and General and Specialist Surgery of University of Campania “Luigi Vanvitelli.”

All procedures in the study were designed in accordance with the Helsinki Declaration of Principles, the Italian National Legislation. This study was approved by the Ethics Committee of the University of Campania “Luigi Vanvitelli”, which formally approved the study (Ethical Committee id codes: 0028204/i, approved on 04 October 2021).

Written informed consent was obtained from a legal guardian for all participants < 18 years.

### Macrophages primary culture

Macrophages were isolated from the peripheral blood mononuclear cells (PBMCs) obtained by density gradient centrifugation (Ficoll 1.077 g/mL, HiSep™ Density Gradient Cell Separation Media, HiMedia Laboratories GmbH, Modautal, Germany). Cells were diluted at 1 × 10^6^ cells/mL in the culture medium α-Minimal Essential Medium (α-MEM) (Lonza, Verviers, Belgium) supplemented with 10% fetal bovine serum (FBS) (Euroclone, Siziano, Italy), 100 IU/mL penicillin, and 100 g/mL streptomycin and L-glutamine (Gibco Limited, Uxbridge, UK) and then plated in 24-well Cell Culture Multiwell. PBMCs were cultured for 15 days in presence of 25 ng/mL recombinant human macrophage colony-stimulating factor (rh-MCSF) (Peprotech, London, UK) in order to obtain fully differentiated macrophages - and cultured at 37 °C in a humidified atmosphere with 5% CO2. Culture medium was replaced twice a week.

At day 15, after complete differentiation, macrophages were treated with a selective CB2 agonist (JWH-133), a CB2 inverse agonist (AM630), a TRPV1 agonist (Resinferatoxin, RTX), and a TRPV1 antagonist (I-RTX). After 24 h of drugs exposure, cells were collected and labelled for proteins extraction, and supernatants for Enzyme Linked Immunosorbent Assay (ELISA) and Iron Assay.

### Drugs and treatments

JWH-133, AM630, RTX and I-RTX were purchased from Tocris (Avonmouth, UK), and dissolved in DMSO. Final concentration of DMSO in cell culture was 0.01%.

Fully differentiated macrophages were treated with JWH-133[100 nm], AM630 [10µM], RTX [5µM] and I-RTX [2,5 µM] for 24 h. These final drug concentrations were determined based on the results of a MTT assay (Supplementary Table S2; Supplementary Table S3), selecting the concentrations that ensured optimal biological activity without affecting cell viability.

Non-treated cultured cells were maintained in incubation media during the same treatment time with or without vehicle (DMSO 0.01%).

### Protein extraction and western blot

For the protein extraction from SCD and HD macrophages, radio-immunoprecipitation assay (RIPA) Lysis Buffer (Millipore, Burlington, USA) was used by following the manufacturer’s instructions. Extracted proteins were quantified through Bradford dye-binding method (Bio-rad, Hercules, CA, USA) and then used to perform Western blot experiments.

The membranes were incubated overnight at 4 °C with the following antibodies: anti-CD86 (1:500, Rabbit, Elabscience, Texas USA), anti-CCR7 antibody (1:500, Rabbit, Elabscience, Texas USA), anti-CD206 (1:200, Mouse, Santa Cruz Biotechnology, Texas USA), anti-pSTAT6 antibody (1:500, Rabbit, Elabscience, Texas USA), anti-FPN-1 antibody (1:1000, Rabbit, Novus Biologicals, Italy), anti-TfR1 antibody (1:1000, Rabbit, Abcam, Cambridge UK), anti-DMT1 (1:100, Mouse, Santa Cruz Biotechnology, Texas USA), anti-CB2 (1:500, Rabbit, Elabscience, Texas USA), and anti-TRPV1 (1:1000, Rabbit, Novus Biologicals, Italy).

Reactive bands on the membrane were detected by chemiluminescence (Clarity max Western ECL Substrate, Biorad, California USA) on CHEMIDOC Bio-Rad (BioRad Hercules, California USA). A mouse monoclonal anti β-Actin antibody (1:500, Mouse, Santa Cruz Biotechnology, Texas USA) was used as housekeeping protein to check the comparable protein loading. Images were captured, stored, and analyzed using Image Lab.Ink 6.1 software.

### ELISA (Enzyme Linked Immunosorbent Assay)

Several ELISA were performed to evaluate the concentration of IL-6, TNF-α, IL-10, IL-5and Hepcidin, using commercially available Human ELISA Kits (Invitrogen by Thermo Fisher, Waltham, MA, USA) and following the manufacturer’s instructions. Briefly, standards and supernatants were pipetted in duplicate into the wells of a microplate, coated with monoclonal antibodies specific to the cytokines of interest. After the plate was washed, enzyme-linked polyclonal antibodies specific for IL-6, TNF-α, IL-10, IL-4, and Hepcidin were added to the wells.

The optical density (OD) was measured by DAS Italy plate reader (DAS Italy, Palombara Sabina - Italia) at a wavelength suggested by the protocol (usually at 450 nm). The concentrations (pg/mL) were determined against a standard concentration curve.

### Iron assay

To determine iron concentration, the Iron Assay Kit (Abcam, Cambridge, UK) was used according to the manufacturer’s protocol. Briefly, standards and supernatants were pipetted in duplicate into the wells of a microplate and incubated with an acidic buffer to allow iron release. Then, we added an iron probe and incubated the microplate at 37 °C for 60 min, protected from light. Released iron reacted with the chromogen resulting in a colorimetric (593 nm) product, proportional to the iron amount. The optical density was measured at a wavelength of 593 nm by DAS Italy plate reader. By using a standard concentration curve, Iron (II) and Total Iron (II + III) contents of the samples (nmol/µL) were determined. Iron (III) content was calculated by using the formulae: Iron (III) = Total Iron (II + III) − Iron (II).

### Statistical analysis

Statistical analyses on data derived from Western Blot, ELISA and Iron Assays performed to compare SCD samples with HD samples, we performed an unpaired t-test.

For statistical analyses on data derived from Western Blot, ELISA and Iron Assays performed to compare the effects of the two different treatments (JWH-133 and AM630) on SCD macrophages compared with NT cells, we firstly performed a Shapiro-Wilk Normality test to asses whether the distribution of our samples were normal or not. For samples with a normal distribution, we employed the one way ANOVA test followed by Tukey HSD as post hoc. For samples that did not show a normal distribution, we employed a Kruskal-Wallis Test, followed by the Dunn’s Test as post hoc.

To perform statistical analysis we used GraphPad Prism (Version 8.4.2).

Each experiment was performed on samples deriving from three different subjects and data expressed as mean ± SD.

A p value ≤ 0.05 was considered statistically significant.

## Supplementary Information

Below is the link to the electronic supplementary material.

Supplementary Material 1

Supplementary Material 2

Supplementary Material 3

Supplementary Material 4

Supplementary Material 5

Supplementary Material 6

Supplementary Material 7

Supplementary Material 8

## Data Availability

The data presented in this study are available in this article (and supplementary material).
