# Supplementary figures and images for: CB2 and TRPV1 receptors in inflammatory state of macrophages from sickle cell anemia pediatric/young adults
Source: Sci Rep. 2025 Aug 8;15:29040. doi: 10.1038/s41598-025-15028-2 (PMC12334692; doi:10.1038/s41598-025-15028-2)

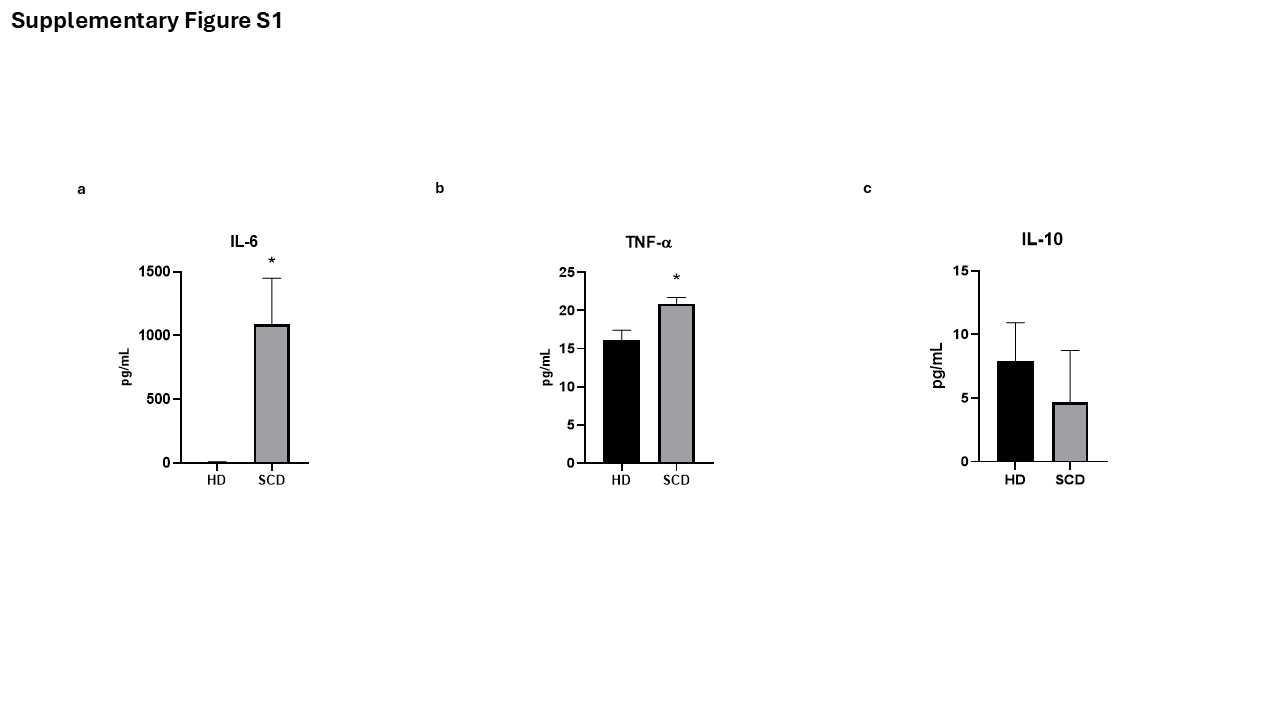

Supplement: Supplementary file 1 — Supplementary Material 1 [file 41598_2025_15028_MOESM1_ESM.tif]

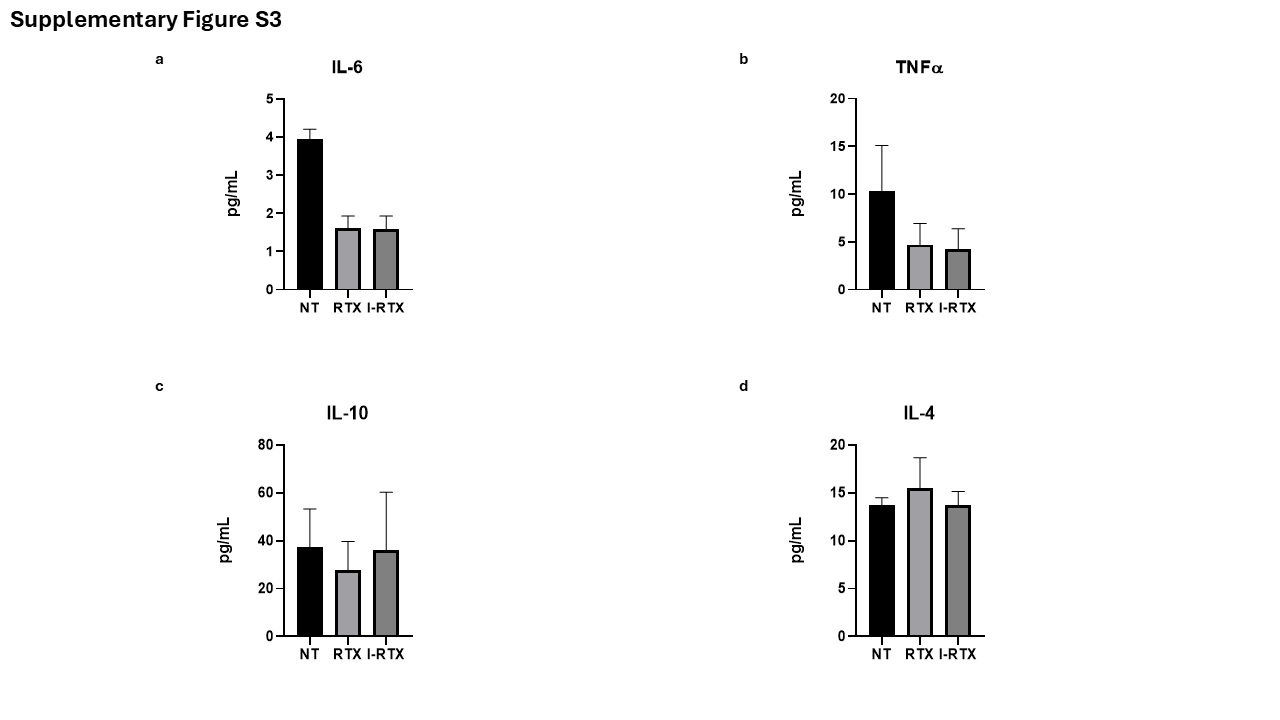

Supplement: Supplementary file 2 — Supplementary Material 2 [file 41598_2025_15028_MOESM2_ESM.tif]

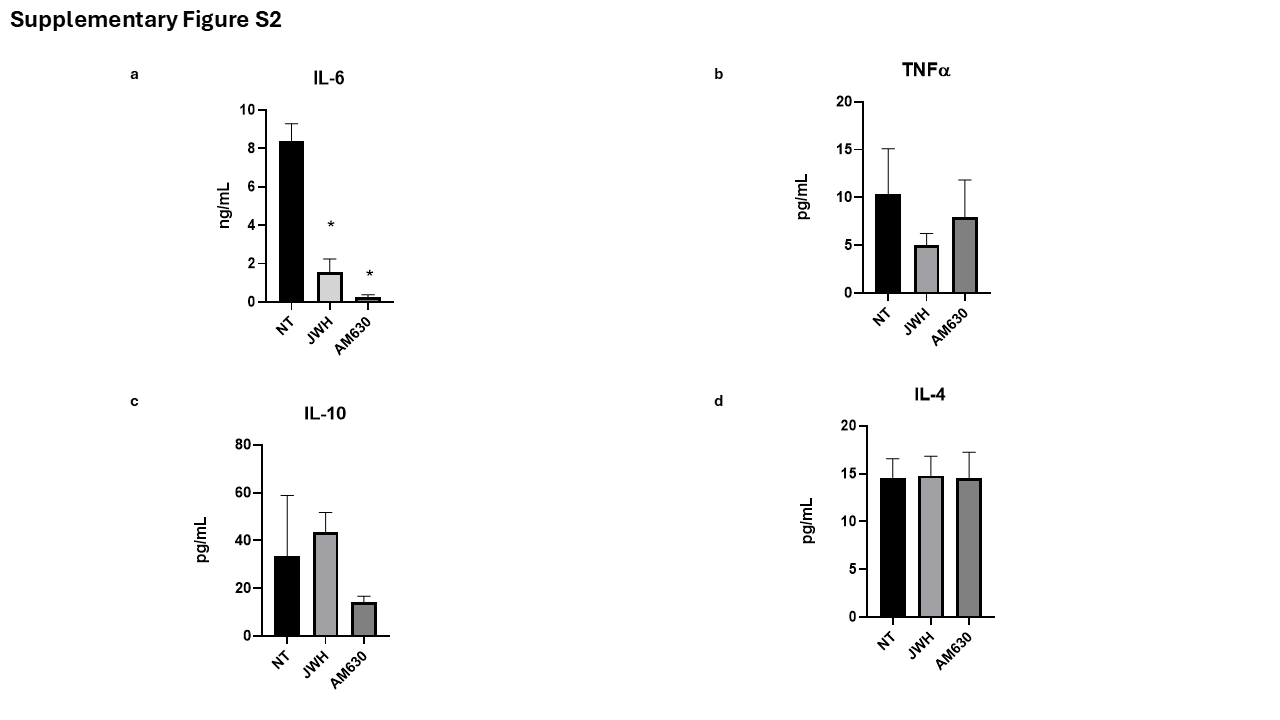

Supplement: Supplementary file 4 — Supplementary Material 4 [file 41598_2025_15028_MOESM4_ESM.tif]
