# Supplementary material for: CB2 and TRPV1 receptors in inflammatory state of macrophages from sickle cell anemia pediatric/young adults
Source: Sci Rep. 2025 Aug 8;15:29040. doi: 10.1038/s41598-025-15028-2 (PMC12334692; doi:10.1038/s41598-025-15028-2)
Supplement: Supplementary file 3 — Supplementary Material 3 [file 41598_2025_15028_MOESM3_ESM.docx]

| N=16 (Hb S/S) | |
| --- | --- |
| Age | 9a5m (2y2m-23y2m) |
| HbS | 73.4% (56-74.7) |
| Ferritin | 274 ng/mL (26-1287) |
| HU | 24.5mg/kg/day (16-27) |
| Antimicrobial Prophyaxis | 10Y – 6N |
| Hb | 10.9 g/dL (7.9-11.4) |
| Reticulocytes | 265x10^3^/µL (140-440) |
| WBC | 8800/µL (1330-11900) |
| PLT | 295x10^3^ /µL (118-480) |
| VOC (prior 6 months) | 1 (0-3) |
| Hospitalization (prior 6 months) | 0.2 (0-1) |
| Infections (prior 6 months) | 2 (0-4) |

**Supplementary Table 1. Clinical characteristics of SCD patients.** This table shows the clinical characteristics of 16 enrolled SCD patients in the study.
