# Supplementary material for: CB2 and TRPV1 receptors in inflammatory state of macrophages from sickle cell anemia pediatric/young adults
Source: Sci Rep. 2025 Aug 8;15:29040. doi: 10.1038/s41598-025-15028-2 (PMC12334692; doi:10.1038/s41598-025-15028-2)
Supplement: Supplementary file 5 — Supplementary Material 5 [file 41598_2025_15028_MOESM5_ESM.docx]

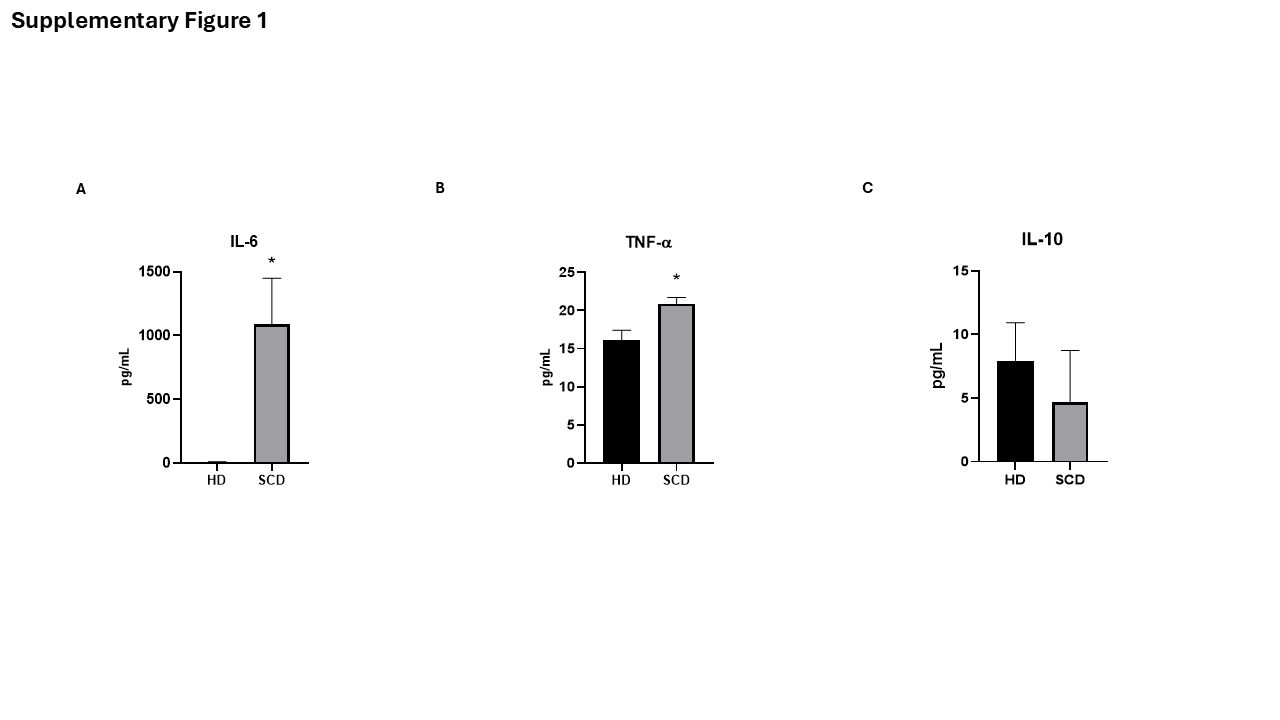


**Supplementary Figure 1. Inflammatory profiles in macrophages derived from SCD pediatric/young patients.** Evaluation of IL-6 (**A**), TNF-α (**B**), and IL-10 (**C**) release from sickle cell disease (SCD) macrophages compared to healthy donors’ (HD) macrophages, revealed through an enzyme-linked immunosorbent assay (ELISA). The graphs show interleukin levels (pg/mL) as the mean ± standard deviation (SD). An unpaired *t*-test was performed for statistical analysis. *, *p* ≤ 0.05 compared to HD.


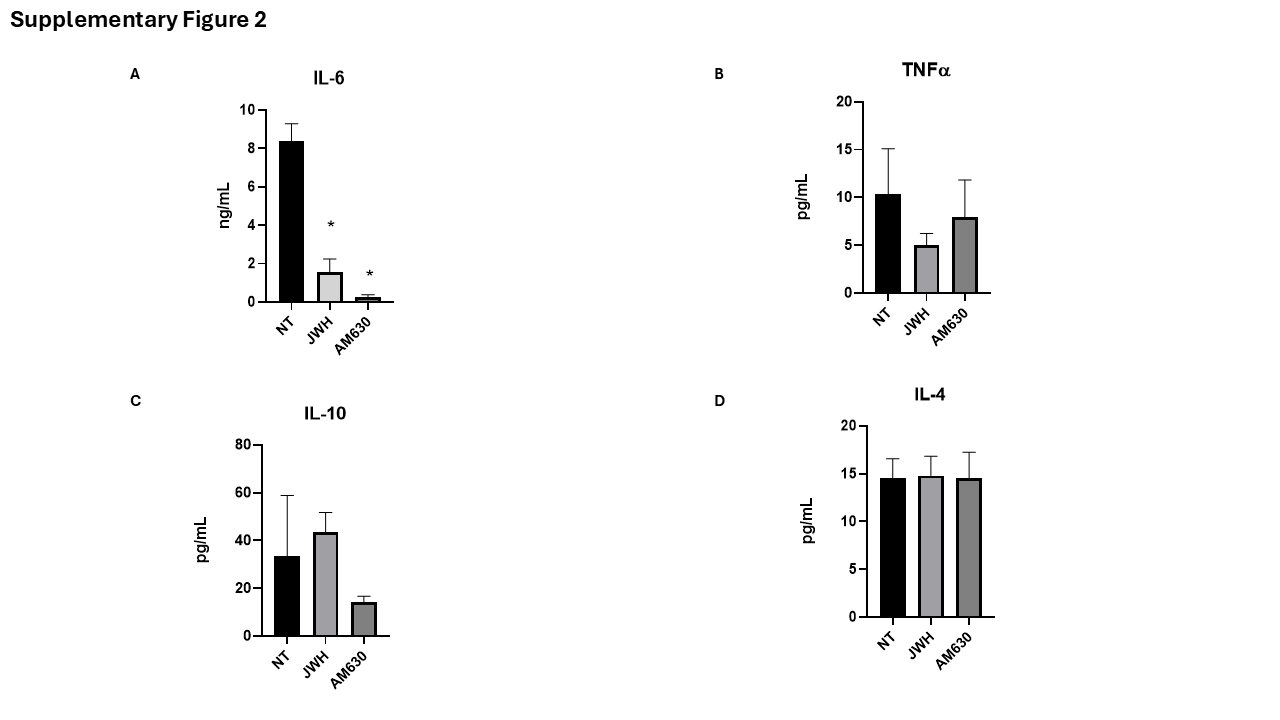


**Supplementary Figure 2. Effects of CB2 stimulation on inflammatory state in macrophages derived from SCD pediatric/young patients.** Evaluation of IL-6 (**A**), TNF-α (**B**), IL-10 (**C**), IL-4 (**D**) release from sickle cell disease (SCD) macrophages treated with JWH-133 and AM630 compared to non-treated (NT) macrophages, revealed through an enzyme-linked immunosorbent assay (ELISA). The graphs show interleukin levels (pg/mL) as the mean ± standard deviation (SD). For statistical analysis, a Shapiro-Wilk Normality test was used to asses whether the distribution of our samples were normal or not. All samples showed normal distribution, and we performed one way ANOVA test followed by Tukey HSD as post hoc. *, *p* ≤ 0.05 compared to NT.


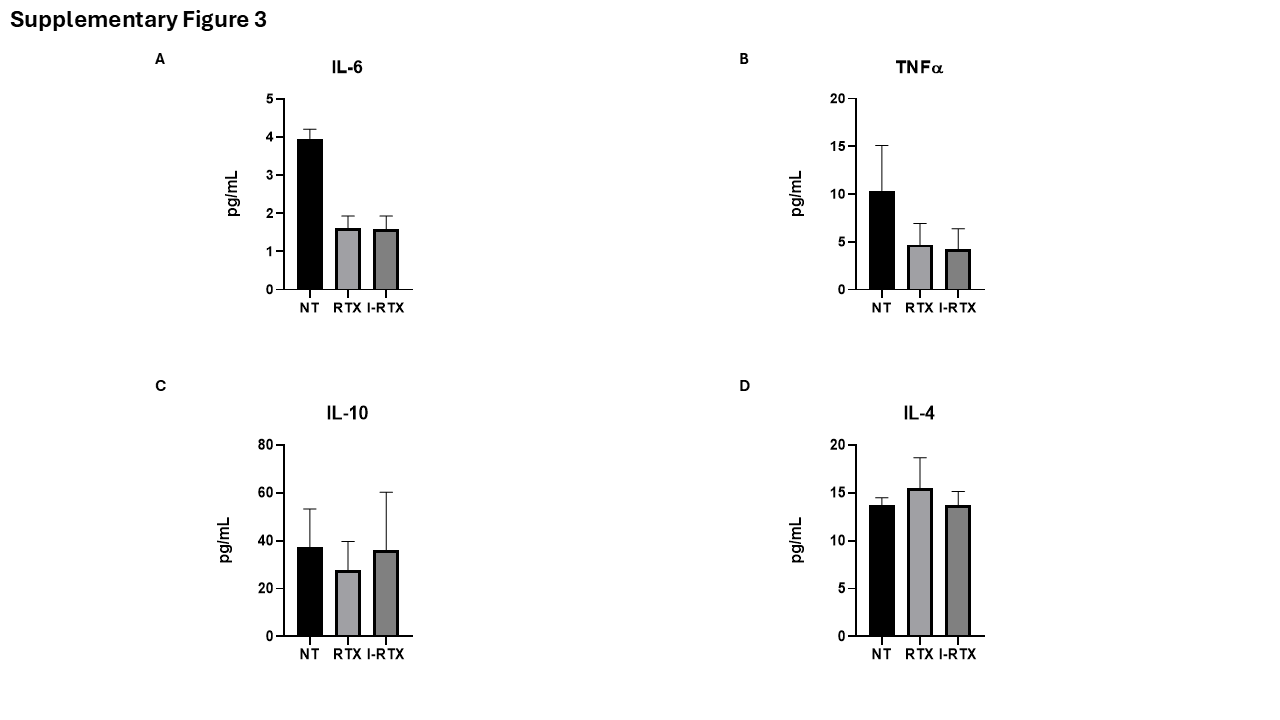


**Supplementary Figure 3. Effects of TRPV1 stimulation on inflammatory state in macrophages derived from SCD pediatric/young patients.** Evaluation of IL-6 (**A**), TNF-α (**B**), IL-10 (**C**), IL-4 (**D**) release from sickle cell disease (SCD) macrophages treated with JWH-133 and AM630 compared to non-treated (NT) macrophages, revealed through an enzyme-linked immunosorbent assay (ELISA). The graphs show interleukin levels (pg/mL) as the mean ± standard deviation (SD). For statistical analysis, a Shapiro-Wilk Normality test was used to asses whether the distribution of our samples were normal or not. For samples with normal distribution (**B**, **C**, **D**), we performed one way ANOVA test followed by Tukey HSD as post hoc. For samples without a normal distribution (**A**), we employed a Kruskal-Wallis Test, followed by the Dunn’s Test as post hoc.
