# Supplementary material for: CB2 and TRPV1 receptors in inflammatory state of macrophages from sickle cell anemia pediatric/young adults
Source: Sci Rep. 2025 Aug 8;15:29040. doi: 10.1038/s41598-025-15028-2 (PMC12334692; doi:10.1038/s41598-025-15028-2)

Supplementary Uncropped WB Images

Figure 1

a

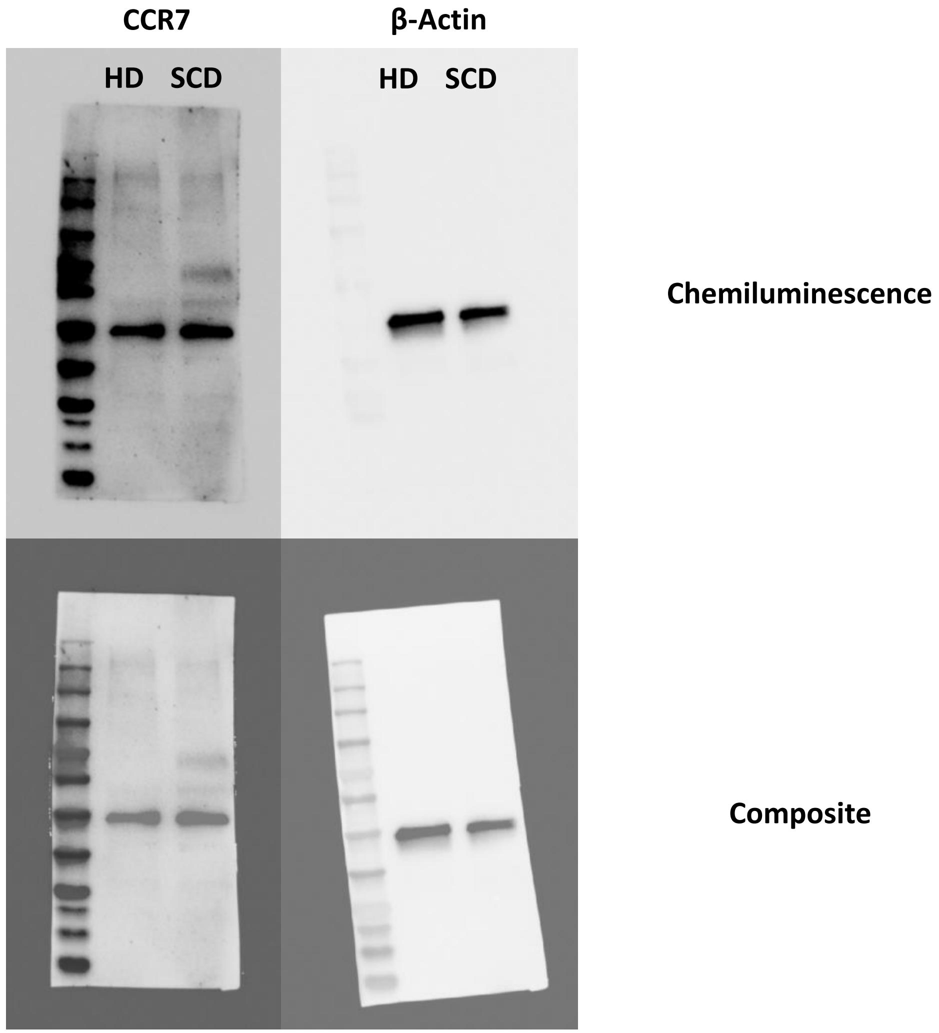

b

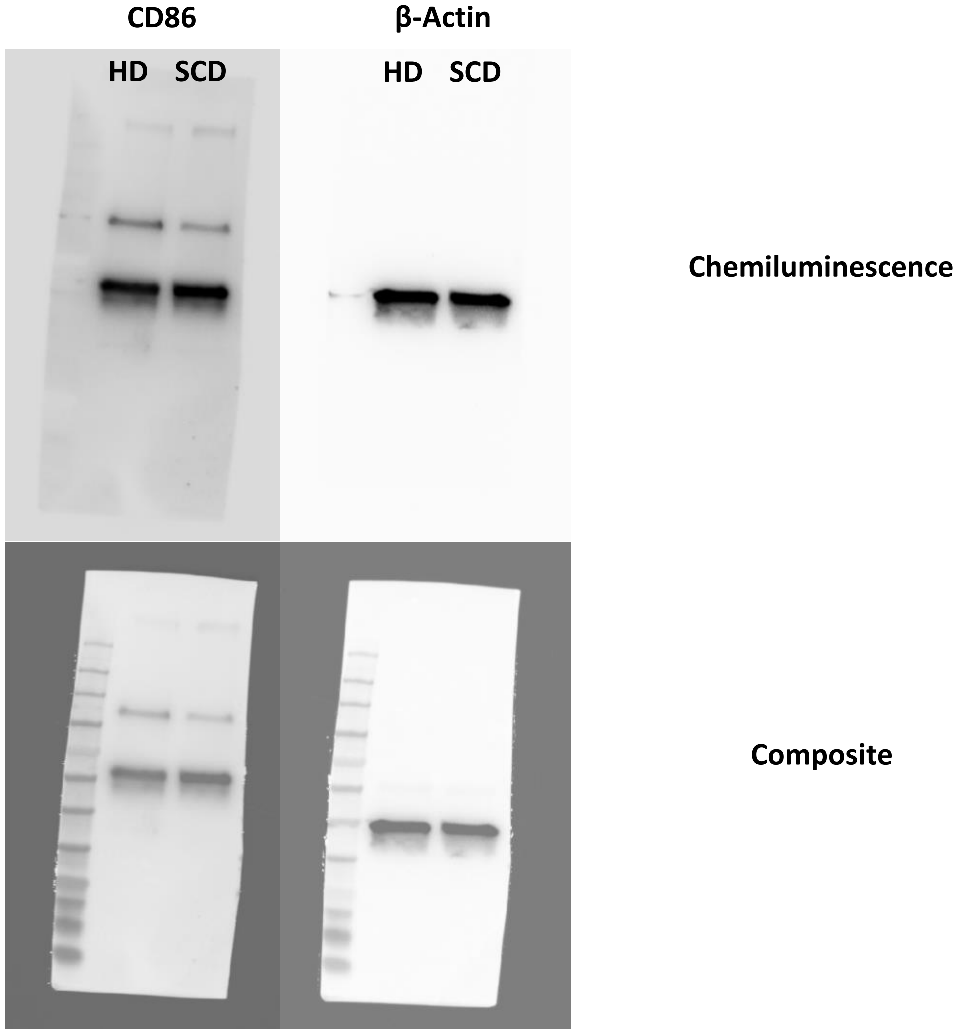

Supplementary Uncropped WB Images

Figure 1

c

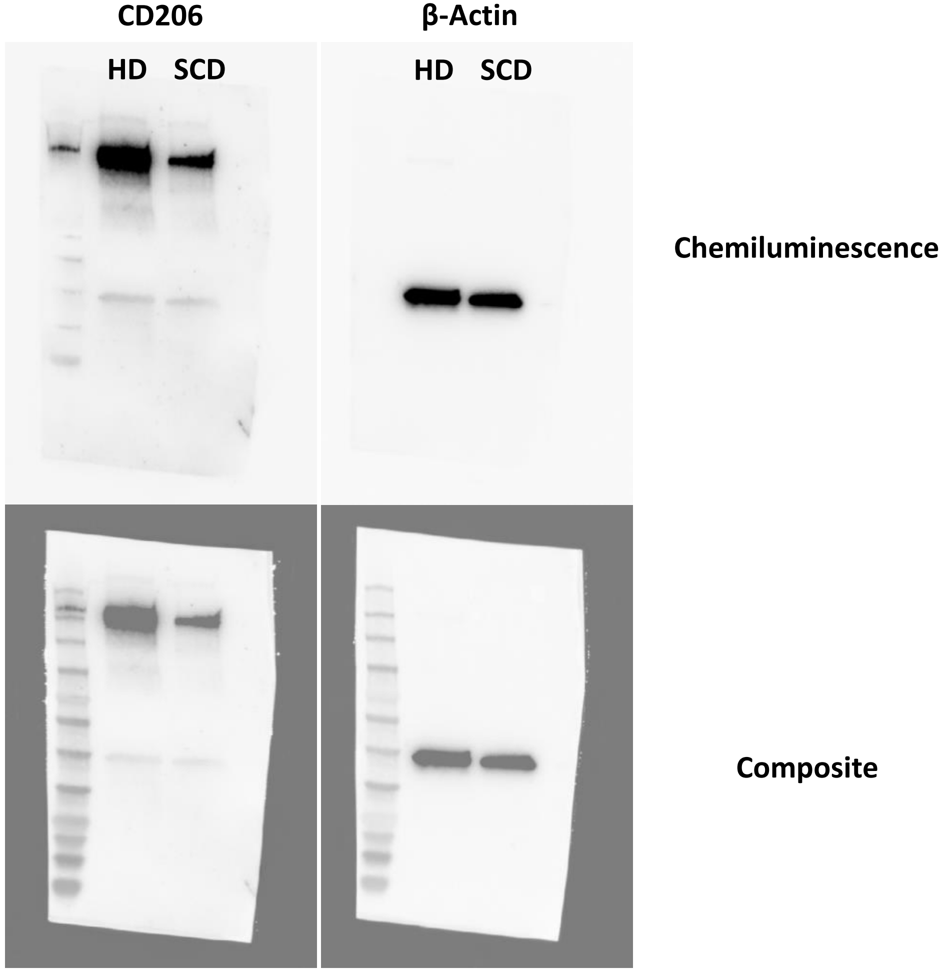

d

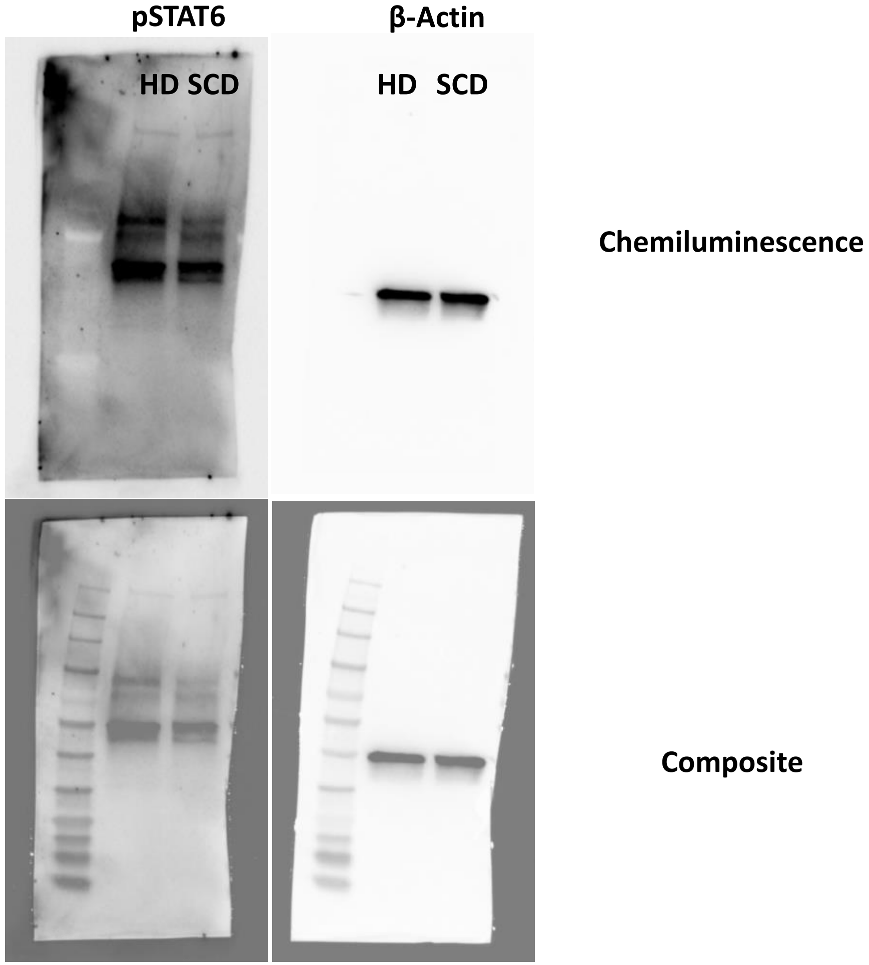

Supplementary Uncropped WB Images

Figure 2

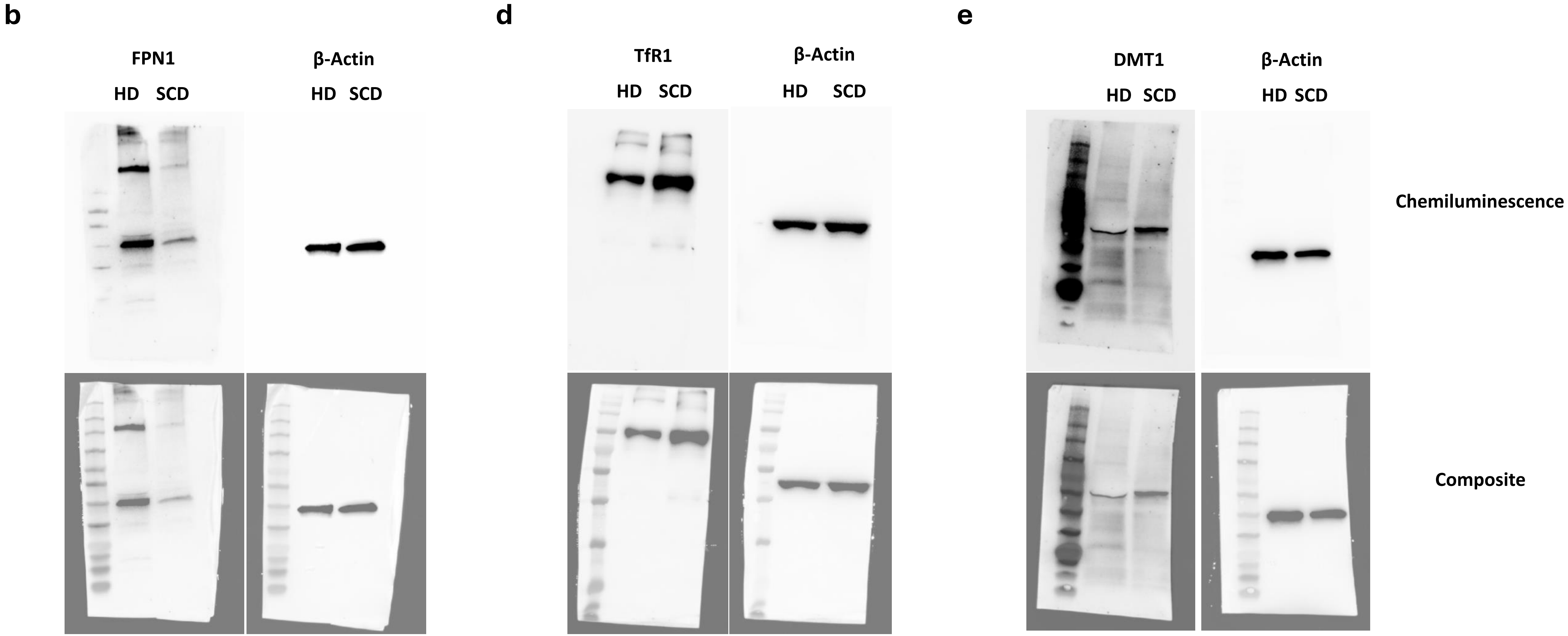

Supplementary Uncropped WB Images

Figure 3

a

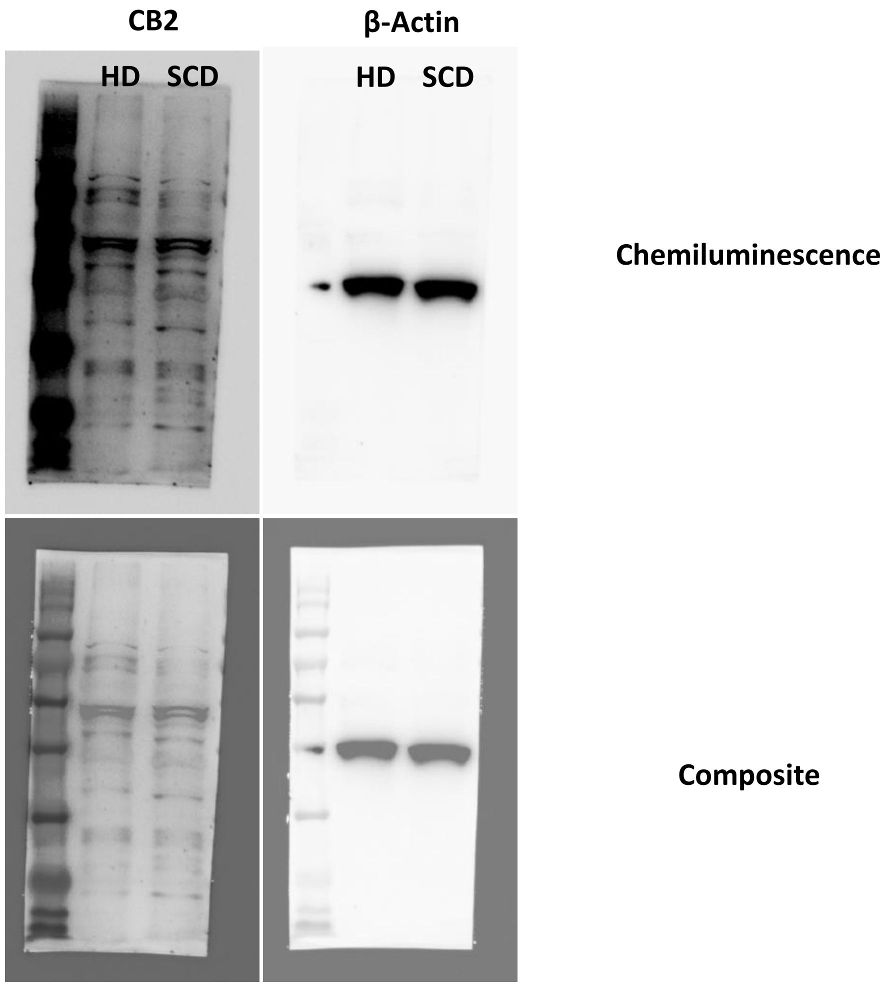

b

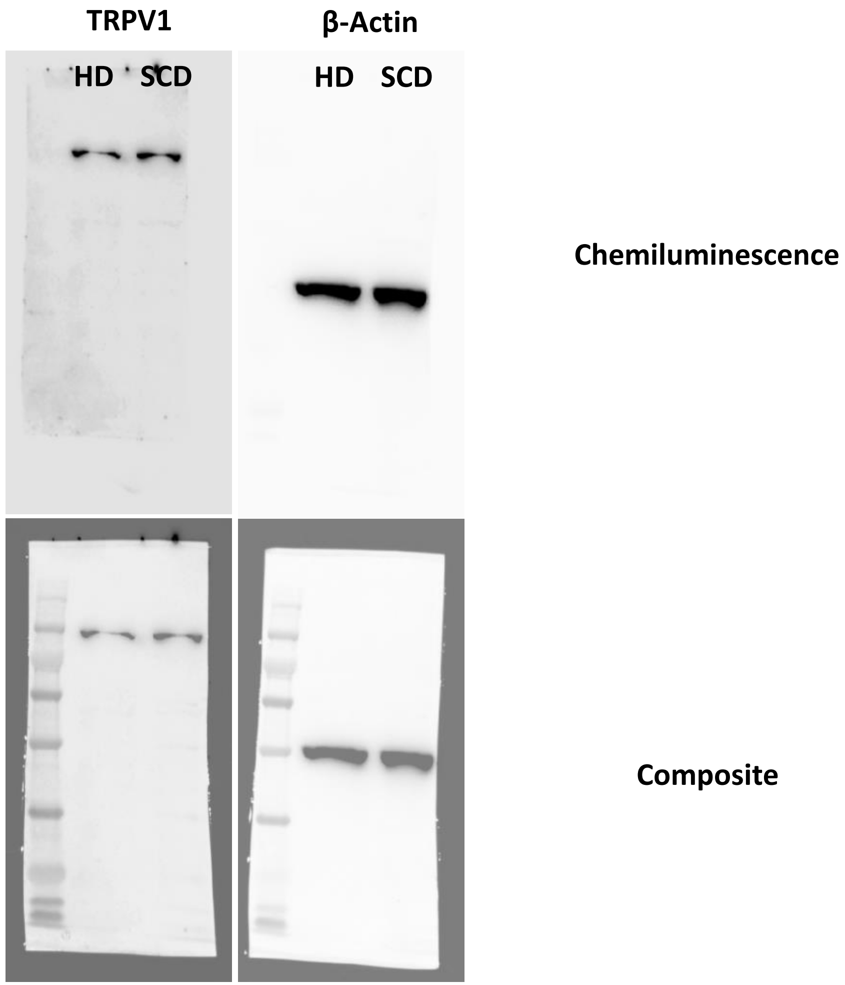

Supplementary Uncropped WB Images

Figure 4

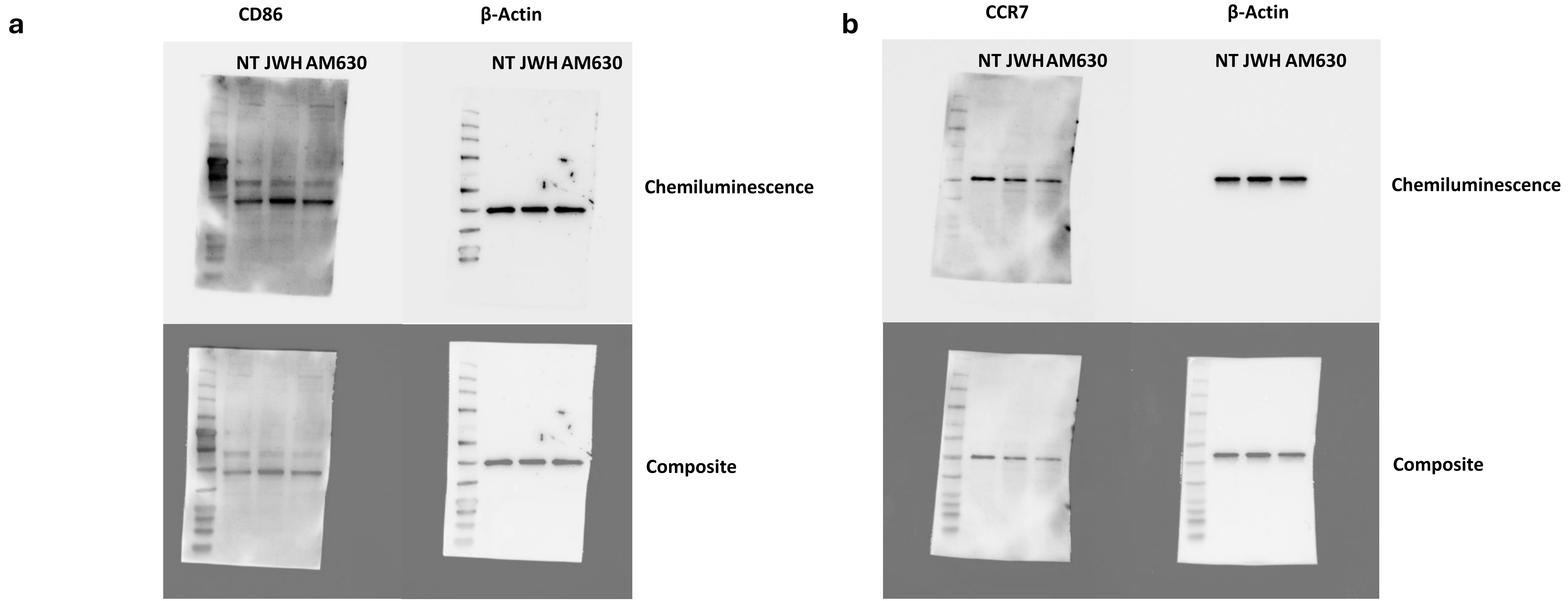

Supplementary Uncropped WB Images

Figure 4

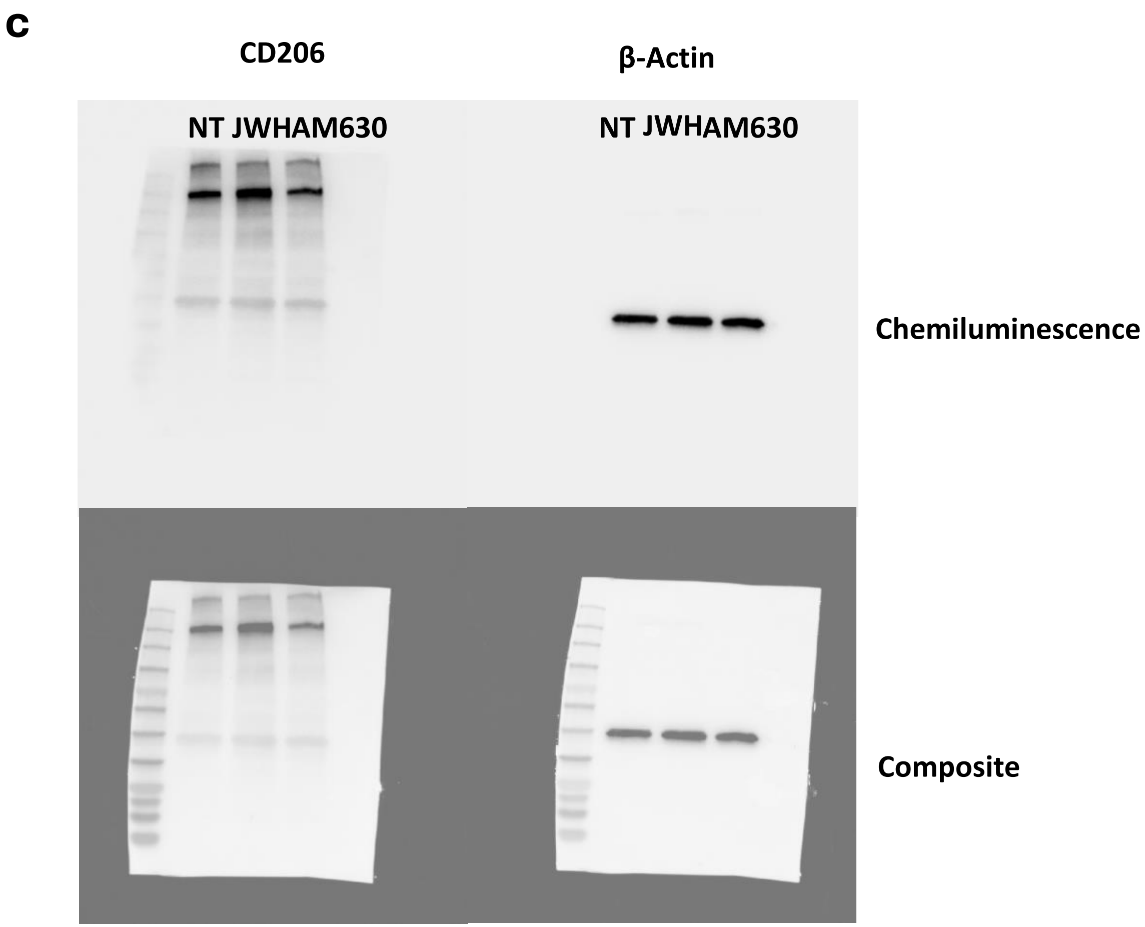

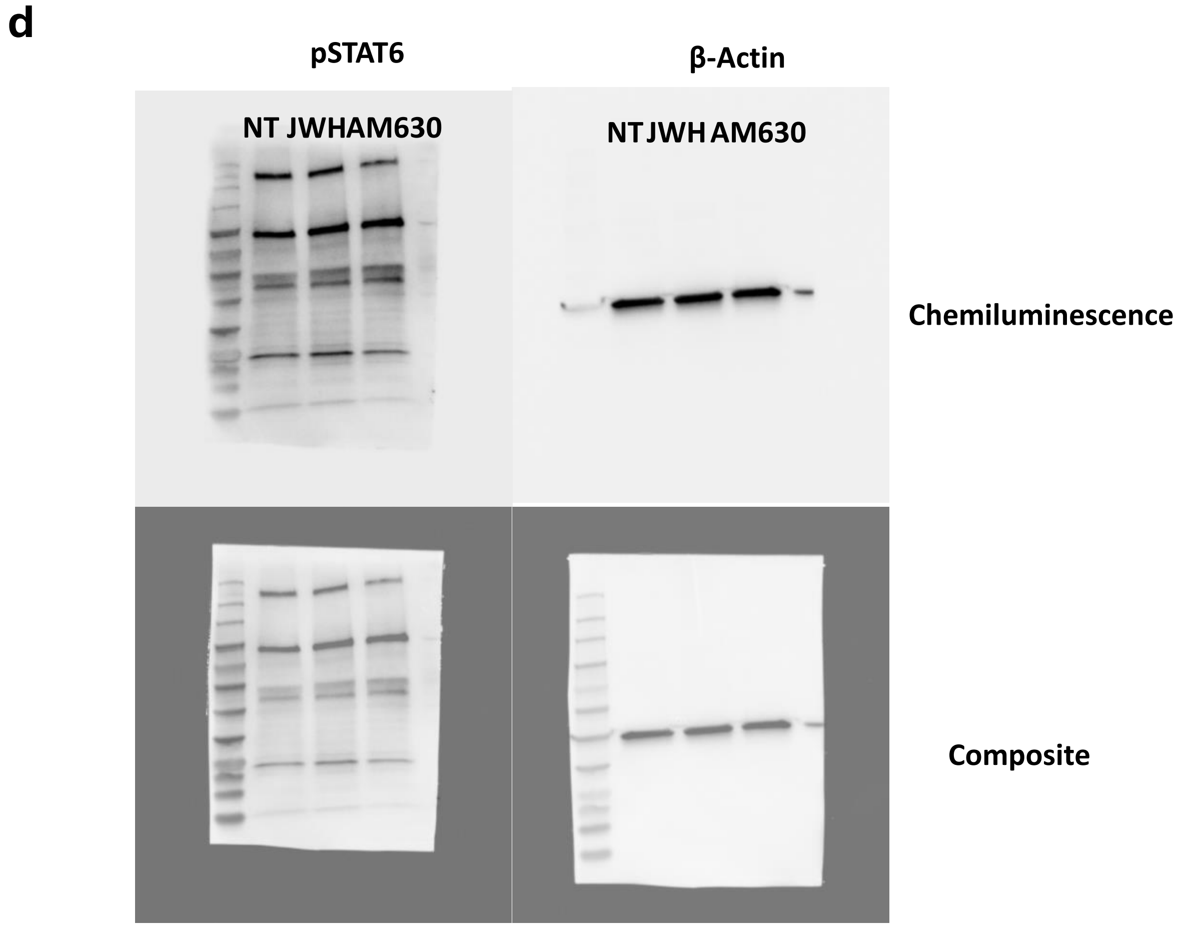

Supplementary Uncropped WB Images

Figure 5

a

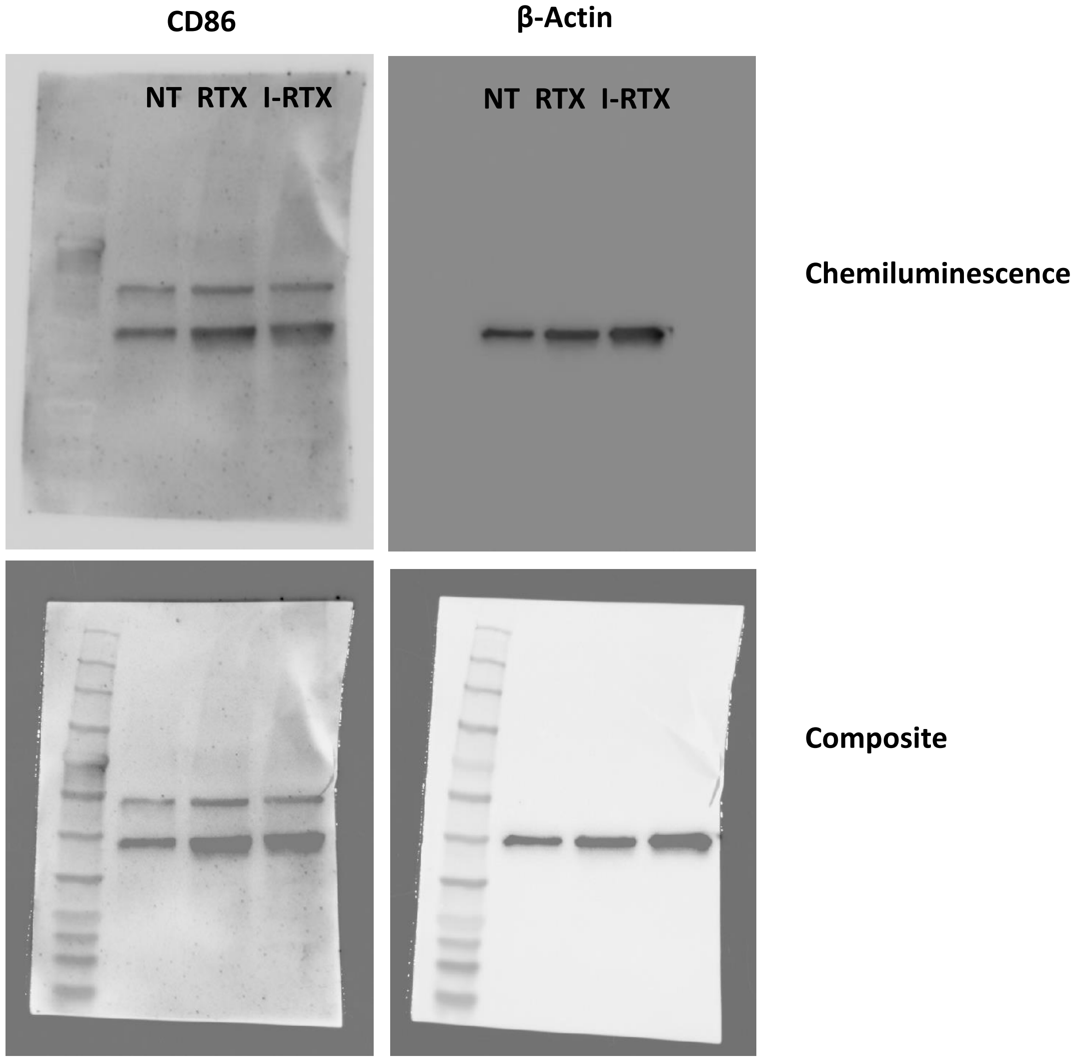

b

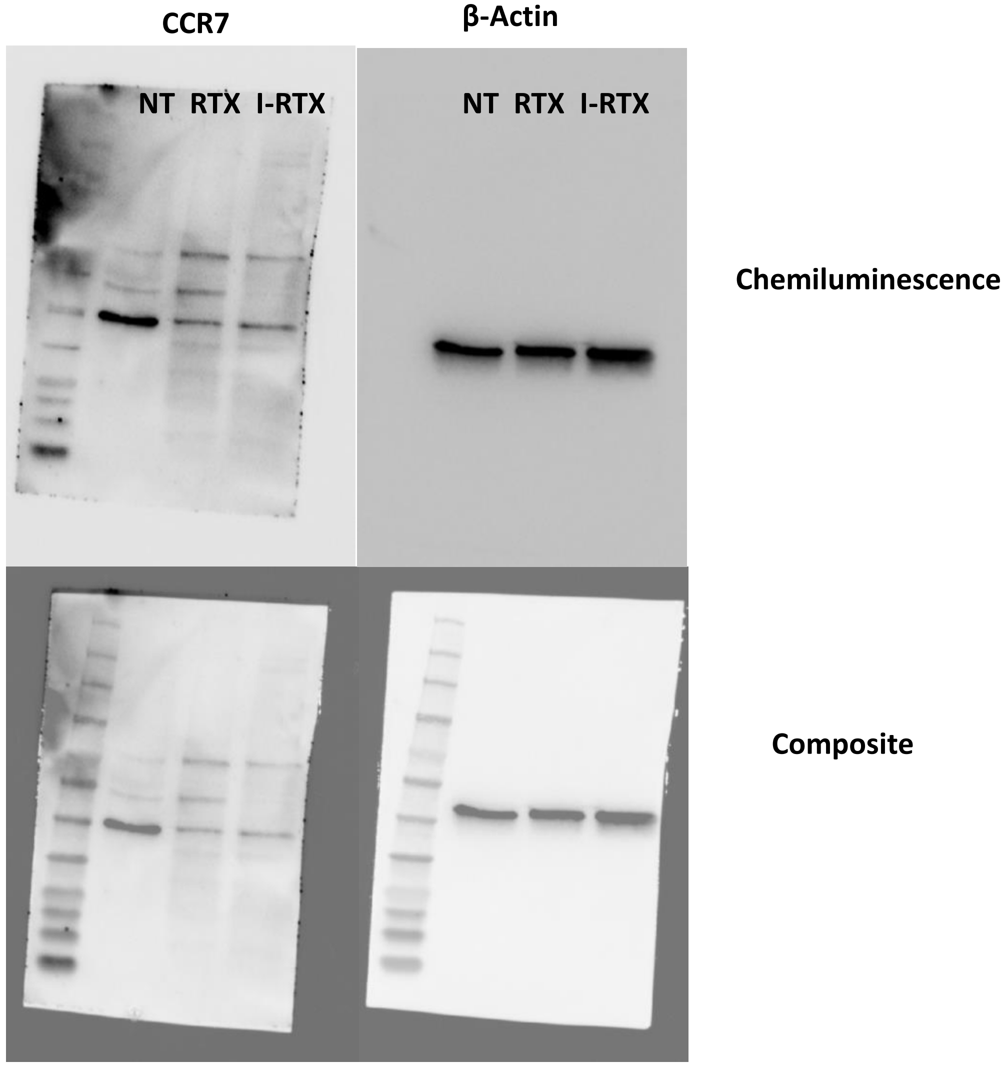

Supplementary Uncropped WB Images

Figure 5

c

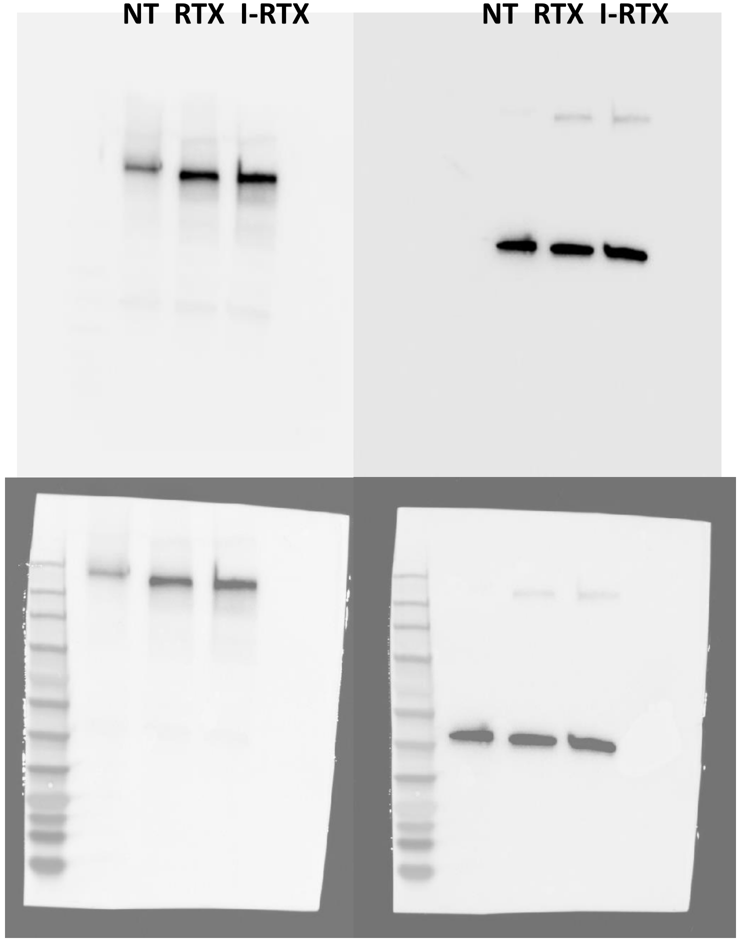

d

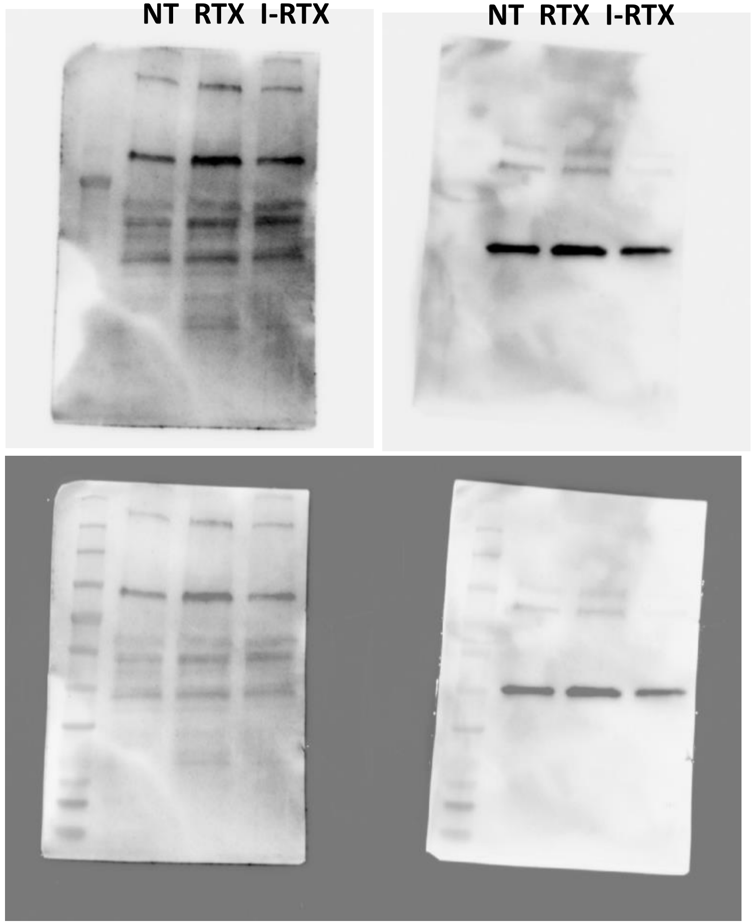

Supplementary Uncropped WB Images

Figure 6

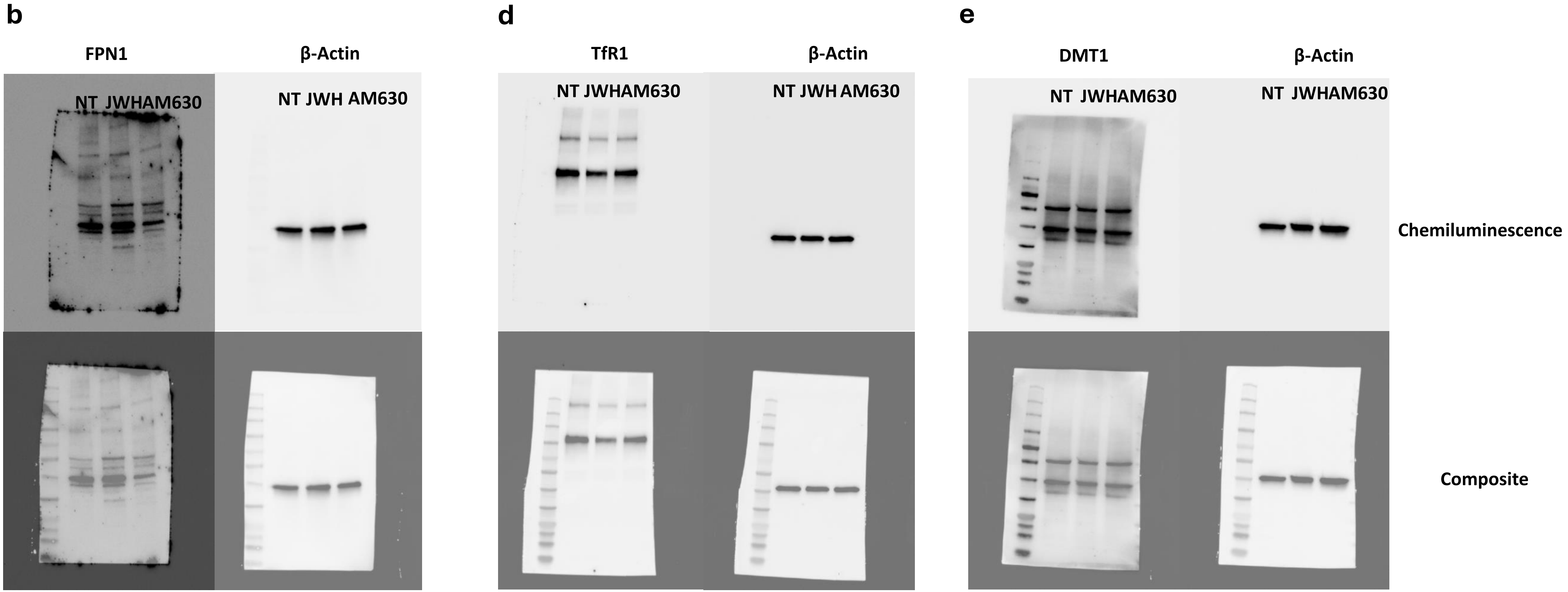

Supplementary Uncropped WB Images

Figure 7

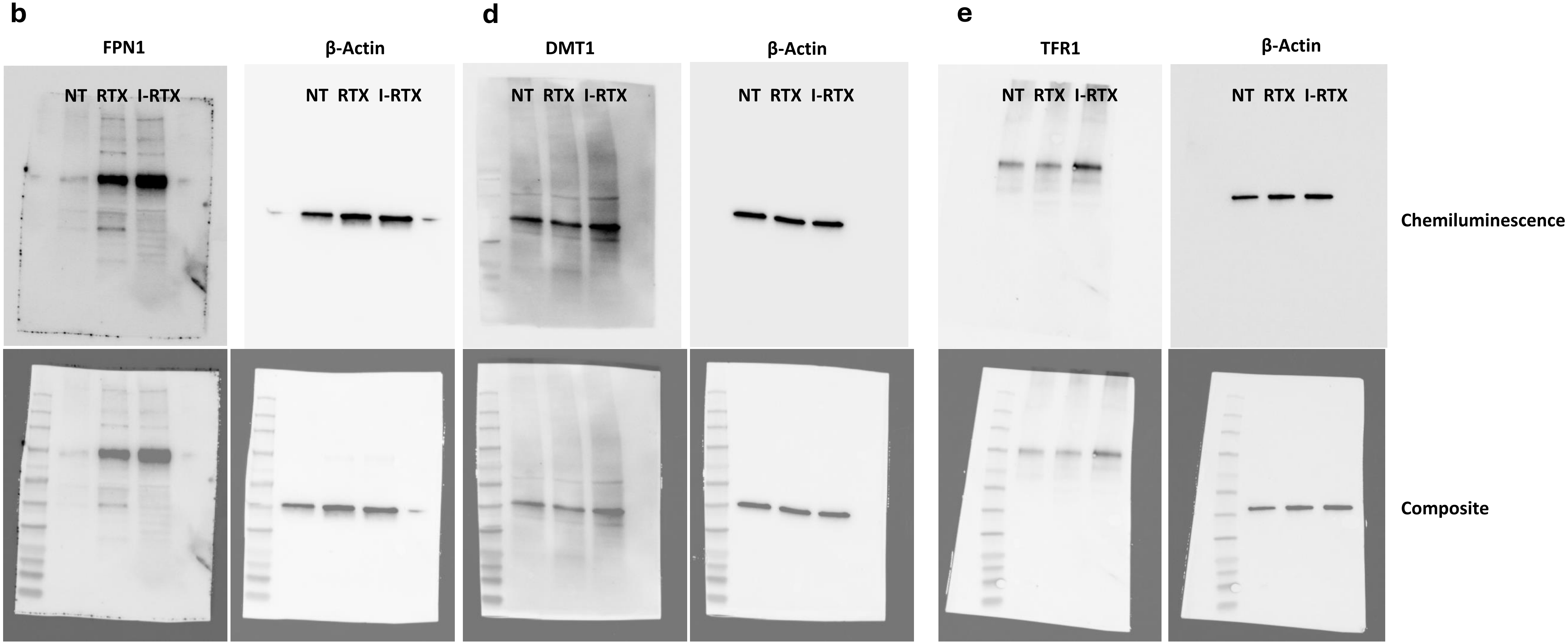

Supplement: Supplementary file 6 — Supplementary Material 6 [file 41598_2025_15028_MOESM6_ESM.pdf]
