# Supplementary material for: CB2 and TRPV1 receptors in inflammatory state of macrophages from sickle cell anemia pediatric/young adults
Source: Sci Rep. 2025 Aug 8;15:29040. doi: 10.1038/s41598-025-15028-2 (PMC12334692; doi:10.1038/s41598-025-15028-2)
Supplement: Supplementary file 8 — Supplementary Material 8 [file 41598_2025_15028_MOESM8_ESM.docx]

**Supplementary Table S3**

| A) Cell Survival Rate (%) – RTX | | |
| --- | --- | --- |
| NT | | 100 ± 0,12 |
| RTX [1 µM] | | 98,8 ± 0,02 |
| RTX [2,5 µM] | | 97,6 ± 0,07 |
| RTX [5 µM] | | 94,9 ± 0,01 |
| RTX [10 µM] | | 87,6 ± 0,04 |
| 1. Cell Survival Rate (%) – I-RTX | | |
| NT | 100 ± 0,05 | |
| I-RTX [1 µM] | 94,2 ± 0,14 | |
| I-RTX [2,5 µM] | 91,8 ± 0,08 | |
| I-RTX [5 µM] | 86,0 ± 0,16 | |
| I-RTX [10 µM] | 82,4 ± 0,04 | |

**Supplementary Table S3.** Cell survival rate in Sickle Cell Disease (SCD) patients’ macrophages after treatment with TRPV1 selective agonist, RTX, at different concentrations (1 µM, 2,5 µM, 5 µM, and 10 µM) (**A**) and its antagonist, I-RTX (1 µM, 2,5 µM, 5 µM, and 10 µM) (**B**). The results are presented as the mean percentage ± standard deviation percentage (SD). For statistical analysis, a Shapiro-Wilk Normality test was used to asses whether the distribution of our samples were normal or not. For samples with normal distribution (B), we performed one way ANOVA test followed by Tukey HSD as post hoc. For samples without a normal distribution (A), we employed a Kruskal-Wallis Test, followed by the Dunn’s Test as post hoc. *, p ≤ 0.05 compared to NT**.**
